# Evaluation of the Performance of Generative AI Large Language Models ChatGPT, Google Bard, and Microsoft Bing Chat in Supporting Evidence-Based Dentistry: Comparative Mixed Methods Study

**DOI:** 10.2196/51580

**Published:** 2023-12-28

**Authors:** Kostis Giannakopoulos, Argyro Kavadella, Anas Aaqel Salim, Vassilis Stamatopoulos, Eleftherios G Kaklamanos

**Affiliations:** 1 School of Dentistry European University Cyprus Nicosia Cyprus; 2 Information Management Systems Institute, ATHENA Research and Innovation Center Athens Greece; 3 School of Dentistry Aristotle University of Thessaloniki Thessaloniki Greece; 4 Mohammed Bin Rashid University of Medicine and Health Sciences Dubai United Arab Emirates

**Keywords:** artificial intelligence, AI, large language models, generative pretrained transformers, evidence-based dentistry, ChatGPT, Google Bard, Microsoft Bing, clinical practice, dental professional, dental practice, clinical decision-making, clinical practice guidelines

## Abstract

**Background:**

The increasing application of generative artificial intelligence large language models (LLMs) in various fields, including dentistry, raises questions about their accuracy.

**Objective:**

This study aims to comparatively evaluate the answers provided by 4 LLMs, namely Bard (Google LLC), ChatGPT-3.5 and ChatGPT-4 (OpenAI), and Bing Chat (Microsoft Corp), to clinically relevant questions from the field of dentistry.

**Methods:**

The LLMs were queried with 20 open-type, clinical dentistry–related questions from different disciplines, developed by the respective faculty of the School of Dentistry, European University Cyprus. The LLMs’ answers were graded 0 (minimum) to 10 (maximum) points against strong, traditionally collected scientific evidence, such as guidelines and consensus statements, using a rubric, as if they were examination questions posed to students, by 2 experienced faculty members. The scores were statistically compared to identify the best-performing model using the Friedman and Wilcoxon tests. Moreover, the evaluators were asked to provide a qualitative evaluation of the comprehensiveness, scientific accuracy, clarity, and relevance of the LLMs’ answers.

**Results:**

Overall, no statistically significant difference was detected between the scores given by the 2 evaluators; therefore, an average score was computed for every LLM. Although ChatGPT-4 statistically outperformed ChatGPT-3.5 (*P*=.008), Bing Chat (*P*=.049), and Bard (*P*=.045), all models occasionally exhibited inaccuracies, generality, outdated content, and a lack of source references. The evaluators noted instances where the LLMs delivered irrelevant information, vague answers, or information that was not fully accurate.

**Conclusions:**

This study demonstrates that although LLMs hold promising potential as an aid in the implementation of evidence-based dentistry, their current limitations can lead to potentially harmful health care decisions if not used judiciously. Therefore, these tools should not replace the dentist’s critical thinking and in-depth understanding of the subject matter. Further research, clinical validation, and model improvements are necessary for these tools to be fully integrated into dental practice. Dental practitioners must be aware of the limitations of LLMs, as their imprudent use could potentially impact patient care. Regulatory measures should be established to oversee the use of these evolving technologies.

## Introduction

### Background

Artificial intelligence (AI) dental applications and tools have exhibited exponential growth during the past few years, aiming to assist health care professionals in providing improved oral health care in a consistent manner. Currently, such tools can support image analysis; the interpretation of radiographs and diagnoses made by neural networks; data synthesis; the provision of information on materials and clinical techniques for improved outcomes; patient record management; other applications in forensic dentistry, orthodontics, periodontology, and endodontics; caries diagnosis; treatment planning; and patient communication and interaction [1]. Using AI technology, clinical questions can be answered on a user’s mobile phone within seconds, and continuing educational updates can be constant [1-7]. Through data synthesis, along with risk factors’ and patterns’ identification, AI could potentially assist in the systematic assessment of clinically relevant scientific evidence, which, when judiciously integrated with the dentist’s clinical expertise in addition to the patient’s treatment needs and preferences, may support busy clinicians in overcoming the challenges associated with the implementation of the evidence-based dentistry (EBD) approach to oral health care [8-10]. Thus, AI may be able to promote individualized patient-centered care and bolster a more efficient, reliable, and standardized clinical practice [11].

On November 30, 2022, an exciting technological innovation in AI, Generative AI (GenAI), was introduced through the launch of ChatGPT (OpenAI Inc), a generative pretrained transformer (GPT) that attracted 100 million users within the first 3 months of its launch, a historical number for an internet application [12]. ChatGPT is a large language model (LLM) that uses natural language processing, an area of AI that aims at enabling computers to understand natural language inputs using a variety of techniques, such as machine learning [12,13]. LLMs are neural networks trained on massive amounts of text data from the internet (from Wikipedia, digitized books, articles, and webpages) with the aim of processing and generating coherent, humanlike conversational responses based on the context of the input text (question or prompt) using deep-learning algorithms and advanced modeling [13-15]. Modern LLMs use a neural architecture based on positional encoding and self-attention techniques to identify relationships within the input text and produce meaningful and relevant responses [16]. They can answer follow-up questions, ask for clarifications, challenge incorrect statements, and reject inappropriate requests [15]. Furthermore, LLMs can be fine-tuned by human evaluators to improve their performance on specific tasks or specialized applications, a process that increases their usability, accuracy, and functionality [16,17]. Unlike conventional search engines, the user does not have to browse, select, and click on a website to obtain an answer; instead, the LLM’s output already collates all available and relevant data from its database in a text response, making it a user-friendly, time-efficient, and seemingly reliable tool. The current free-access version of ChatGPT is based on the GPT-3.5 language model, and the newer version, GPT-4, is currently available under the ChatGPT Plus paid subscription. Later, in February 2023, Microsoft launched the Bing Chat AI chatbot, which uses the GPT-4 language model, whereas in March 2023, Google released the Bard chatbot, which was powered initially by Language Model for Dialogue Applications (LaMDA), its proprietary family of LLMs, and later by the Pathways Language Model (PaLM) 2 LLM.

ChatGPT-3.5 and the improved, subscription version ChatGPT-4, compared with their competitors, are easy to use and available to everyone on OpenAI’s website. This widespread accessibility makes these bots a top choice for many users. By contrast, although Bing Chat has its strengths, such as being suitable for research, having live access to the internet, and having access to GPT-4, its limited accessibility is a drawback. Bing Chat has a chat limit of 100 requests per day, which, compared with ChatGPT’s 70 requests per hour, can be a bottleneck in a research study. This, in tandem with its somewhat limited browser compatibility, makes it unsuitable for everyday use. Google Bard also has live access to the internet but is still in its early stages, both technologically and commercially [18,19].

ChatGPT is the most studied LLM so far in education, research, and health care, with promising results and some valid concerns. Benefits in health care clinical practice could include cost saving, documentation, personalized medicine, health literacy, and the streamlining of workflow, whereas in dentistry and oral health care, ChatGPT could be used as a supplementary tool for better diagnosis and decision-making, data recording, image analysis, disease prevention, and patient communication [14,17,20,21]. Rao et al [22] evaluated ChatGPT’s capacity for clinical decision support in radiology through the identification of appropriate imaging modalities for various clinical presentations of breast cancer screening and breast pain and concluded that the integration of such AI tools into the clinical workflow is feasible and efficient. The coupling of LLMs with EBD seems ideal, as dental professionals can have evidence-based, fact-driven, and patient-specific responses to clinical queries within seconds, an approach that could potentially enable the identification of treatment choices and the decision-making process, lower the chances of mistakes, and enhance personalized dental care and practice efficiency.

The serious concerns raised about different aspects of GenAI technologies include the criteria and goals of the developers, personal data protection and encryption vulnerability, and the validity of the information provided by these models [1,23]. The major question at present is which aspects of GenAI provide real benefits to society and which present potential problems [24,25]. In March 2023, Italy banned the use of ChatGPT owing to privacy concerns, as there was no secure way to protect personal data and financial information could thus potentially be stolen through this technology [26]. However, the ban was lifted after OpenAI met the demands of regulators regarding privacy concerns [27]. ChatGPT is also banned in countries with heavy internet control, such as North Korea, Iran, Russia, and China [26].

Furthermore, there are several considerations regarding the use of GenAI in health care, such as the output’s accuracy; the possibility of unreliable responses, including the risk of hallucination, that is, the presentation of entirely wrong, inaccurate or even harmful responses and fabricated information as real; the risk of biased diagnoses; and ethical and legal issues. Major drawbacks for health-related queries include the limited knowledge database (ChatGPT’s database at the time of the study did not extend beyond 2021), the inability to evaluate the credibility of information retrieval sources, and the inability to integrate external resources outside their databases (eg, scientific journals and textbooks) [1,12,14,20,28-30]. Considering the abovementioned limitations, it seems logical that despite the data set and training provided to these models, they cannot replace unique human intellectual abilities, and users must exercise caution and apply all means of evaluation, validation, and critical thinking to the information received.

### Objectives

This study aimed to compare the performance of currently available GenAI LLMs in answering clinically relevant questions from the field of dentistry by assessing their accuracy against traditional, evidence-based scientific dental resources. The null hypothesis is that there is no difference in comprehensiveness, scientific accuracy, clarity, and relevance among the 4 LLMs and between the 4 LLMs and the evidence-based scientific literature. By conducting this comparative analysis, this study aimed to shed light on the advantages and disadvantages of using LLMs in dental practice and initiate a debate about the role of AI technologies in EBD. This study may be the first to evaluate the clinical use of ChatGPT and similar chatbots as chairside dental assistants to promote EBD and clinical decision-making.

## Methods

### Overview

A total of 20 questions relevant to clinical dentistry were asked to the 4 different LLMs. The questions were regarding common clinical issues related to different dental disciplines (Multimedia Appendix 1). The LLMs tested were (1) ChatGPT model GPT-3.5 (offered for free at the moment), (2) ChatGPT model GPT-4 (offered through ChatGPT Plus under subscription), (3) Google Bard, and (4) Microsoft Bing Chat. These LLMs appear to be the most popular and powerful chatbots in GenAI at the moment.

A pool of questions was developed by the faculty of the School of Dentistry, European University Cyprus, in the disciplines of oral surgery and oral medicine and oral pathology, endodontology, operative dentistry, orthodontics, periodontology, pediatric dentistry, oral and maxillofacial radiology, and prosthodontics. The specialists were asked to provide questions that were clinically relevant and had answers that were supported by strong evidence. The questions used were agreed upon among the authors, through a consensus process, based on the following criteria: (1) they would be of interest to the general dentist; therefore, questions on specific fields that can be answered solely by specialists were not considered; (2) they would cover a broad spectrum of dental procedures performed in routine clinical practice, such as operative dentistry, radiology, prosthodontics, oral surgery, and periodontology; and (3) they would have indisputable, unequivocal answers supported by scientific evidence. This evidence was provided by specialists. They were retrieved mainly from guidelines issued by scientific organizations and academies; consensus statements; textbooks; professional and educational bodies, such as the Federation Dentaire Internationale (FDI) and the American Dental Association (ADA); medical libraries; and a PubMed database search for systematic reviews in high-impact, peer-reviewed scientific journals. All pieces of evidence retrieved clearly addressed the questions and were of the highest quality available [31]. They served as the gold standard with which the LLMs’ responses were compared.

Questions or prompts were written in scientific language using appropriate terminology and were open ended, requiring a text-based response. Each question was asked once to each LLM by one of the authors, with no follow-up questions, rephrasing, or additional explanation in case of the LLM’s inability to answer. It was also not asked for a second time by another author. By simulating scenarios in which oral health care professionals seek immediate assistance with single questions, our study mirrored real-world situations. This approach made it easier to assess how the LLMs could assist dentists in quick, on-demand information retrieval and clarification, a valuable skill in health care practice.

Moreover, limiting interactions to single queries allowed for a more focused evaluation of the LLMs’ ability to provide concise and relevant responses to complex queries without the need for reprompting, meaning that the process can be once-off and not time consuming.

The answer to each question was evaluated and graded by 2 experienced faculty members of the School of Dentistry, European University Cyprus, who were informed that they were grading LLMs’ responses (authors KG and AAS). The first author is a coordinator of operative dentistry courses and holds a graduate degree in advanced education in general dentistry and PhD in operative dentistry. The second author is a coordinator of operative dentistry and critical appraisal of the literature courses and holds a PhD in operative dentistry. The LLMs’ answers were graded 0 (minimum) to 10 (maximum) points against a rubric (Multimedia Appendix 2). The evaluators were blinded to the names of the LLM, as each LLM was referred to by a letter; therefore, they were unaware of which LLM they were grading. The correct answer or “gold standard,” based on which they were asked to evaluate the answers provided by the LLMs, was given to the evaluators and was allocated the maximum grade of 10/10. As the “gold standard” was provided, no other calibration was required. A mixed methods approach (quantitative and qualitative research) was used.

### Qualitative Evaluation

The evaluators were asked to provide a qualitative evaluation of the LLMs’ responses in terms of their scientific accuracy, comprehensiveness, clarity, and relevance in the form of free text. Specifically, they were asked to provide explanatory comments on the LLMs’ answers, which would document their chosen grade and would result from critically comparing the LLMs’ answers with the “gold standard.” In their analysis of the answers, the evaluators could indicate the specific elements that were false, irrelevant, outdated, or contradictory and their effect on clinical practice if they were actually applied by the dentist. Comments could include positive aspects of the answers, for example, stating that the answers were detailed, accurate, and well articulated and addressed the subject sufficiently, as well as negative aspects of the answers, for example, stating that the answers were inaccurate, unclear, or incomplete; did not match the “gold standard”; and, therefore, could not provide relevant and scientifically correct guidance for an evidence-based practice.

### Statistical Analyses

The data were summarized by calculating indices of central tendency (mean and median values) and indices of variability (minimum and maximum values, SDs and SE of mean values, and coefficient of variation). To assess reliability, Cronbach α and intraclass correlation coefficient (ICC) were calculated. To test whether there was a correlation between the scores of the 2 evaluators, Pearson *r* and Spearman ρ were calculated. Furthermore, to test the differences between the scores, Friedman and Wilcoxon tests were performed. All statistical analyses were performed using SPSS (version 29.0; IBM Corp), which was enhanced using the module Exact Tests (for performing the Monte Carlo simulation method) [32]. The significance level in all hypotheses and testing procedures was predetermined at Cronbach α=.05 (*P*≤.05).

### Ethical Considerations

The study does not involve any humans or animals. We have a confirmation certificate of the President of the Institutional Committee on Bioethics and Ethics of the European University Cyprus that no ethical approval is needed for this project.

## Results

### Overview

Table 1 presents the descriptive statistics for the scores given by the 2 evaluators for the answers provided by the 4 LLMs. Both evaluators scored the answers of ChatGPT-4 as the best, followed by the answers of ChatGPT-3.5, Google Bard, and Microsoft Bing Chat.

Multimedia Appendix 3 presents the answers of the LLMs to the 20 questions and a short description of the evidence that was used as the gold standard against which the answers were graded.

The interevaluator reliability, that is, the correlation between the scores given by the 2 evaluators for the answers provided by the 4 LLMs, is presented in Table 2. Pearson *r* and Spearman ρ revealed strong and statistically significant correlations between their scores, suggesting that the answers of the 4 LLMs were evaluated in the same way. Similarly, Cronbach α and ICC suggested high reliability. All Cronbach α values were >.6, and all ICCs were statistically significant (Table 2). Corroborating evidence was provided by Wilcoxon test, which did not detect any statistically significant difference overall between the scores given by the 2 evaluators for the answers provided by the 4 LLMs (Table 2), except for the scores given for the answers provided by ChatGPT-4, between which a marginally statistically significant difference was found (*P*=.049). Therefore, an average score was computed for the scores provided by the 2 evaluators for each LLM.

**Figure 1:**
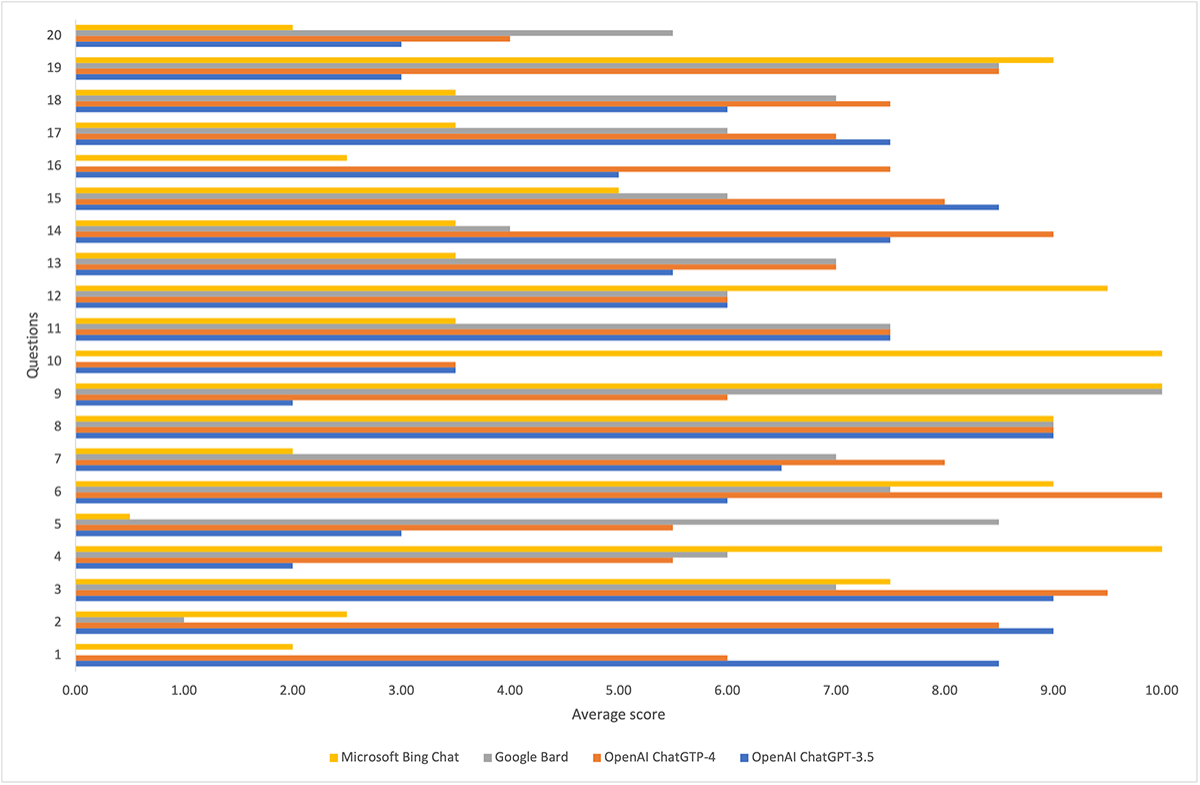
The average scores for the answers provided by the 4 large language models to each question.

Figure 1 presents the average scores for the answers provided by the 4 LLMs to each question. Table 3 presents the descriptive statistics for the average scores for the answers provided by the 4 LLMs. The answers of ChatGPT-4 were scored as the best, followed by the answers of ChatGPT-3.5, Google Bard, and Microsoft Bing Chat.

Friedman test revealed statistically significant differences between the average scores of the 4 LLMs (*P*=.046); therefore, a series of pairwise Wilcoxon tests were performed. According to the Wilcoxon’s test results, a statistically significant difference between the average scores of ChatGPT-3.5 and ChatGPT-4 was noted (*P*=.008), and marginally statistically significant differences were noted between the average scores of ChatGPT-4 and Microsoft Bing Chat (*P*=.049) and between the average scores of ChatGPT-4 and Google Bard (*P*=.045). No other statistical differences were detected between the average scores of the other LLMs (Table 4). On the basis of the aforementioned statistics, the answers that scored the best were from ChatGPT-4 (average score=7.2), followed by those from ChatGPT-3.5 (average score=5.9), Google Bard (average score=5.7), and Microsoft Bing Chat (average score=5.4).

**Table 1:** Descriptive statistics for the scores given by the 2 evaluators for the answers provided by the 4 large language models.

	OpenAI ChatGPT-3.5		OpenAI ChatGPT-4			Google Bard			Microsoft Bing Chat	
	Evaluator 1	Evaluator 2	Evaluator 1	Evaluator 2	Evaluator 1		Evaluator 2	Evaluator 1		Evaluator 2
Minimum	1	2	2	4	0		0	1		0
Median	6	6	8	7	7		7	4		4
Maximum	10	10	10	10	10		10	10		10
Mean (SD; SE)	5.8 (3.2; 0.7)	6.1 (2.3; 0.5)	7.7 (2.1; 0.5)	6.7 (1.9; 0.4)	5.8 (3.4; 0.8)		5.6 (3.1; 0.7)	5.6 (3.5; 0.8)		5.3 (3.5; 0.8)
Coefficient of variance (%)	55.0	38.1	26.9	29.1	59.4		55.9	63.9		66.5

**Table 2:** Correlation between the scores (Pearson *r* and Spearman ρ), Cronbach α, intraclass correlation coefficient (ICC) for the scores, and Wilcoxon *P* value for the scores given by the 2 evaluators for the answers provided by the 4 large language models (LLMs).

LLMs	Pearson *r*	*P* values	Spearman ρ	*P* values	Cronbach α	ICC single	*P* values	ICC average	*P* values	Wilcoxon test *P* value
OpenAI ChatGPT-3.5	0.580	*.007* ^a^	0.620	*.004*	.711	0.561	*.005*	0.719	*.005*	.81
OpenAI ChatGPT-4	0.536	*.01*	0.586	*.007*	.689	0.492	*.006*	0.659	*.006*	*.049*
Google Bard	0.779	*<.001*	0.611	*.004*	.873	0.782	*<.001*	0.877	*<.001*	.75
Microsoft Bing Chat	0.847	*<.001*	0.744	*<.001*	.917	0.850	*<.001*	0.919	*<.001*	.47

^a^Statistically significant values are italicized.

**Table 3:** Descriptive statistics for the average scores to the answers provided by the 4 large language model.

	OpenAI ChatGPT-3.5	OpenAI ChatGPT-4	Google Bard	Microsoft Bing Chat
Minimum	2.0	3.5	0.0	0.5
Median	6.0	7.5	6.5	3.5
Maximum	9.0	10.0	10.0	10.0
Mean (SD; SE)	5.9 (2.4; 0.5)	7.2 (1.8; 0.4)	5.7 (3.1; 0.7)	5.4 (3.4; 0.8)
Coefficient of variance (%)	40.7	25.0	54.4	63.0

**Table 4:** Wilcoxon test *P* value for the average scores for the answers provided by the 4 large language models (LLMs).

LLM	Wilcoxon *P* value
OpenAI ChatGPT-3.5 vs OpenAI ChatGPT-4	*.008* ^a^
OpenAI ChatGPT-3.5 vs Google Bard	.84
OpenAI ChatGPT-3.5 vs Microsoft Bing Chat	.63
OpenAI ChatGPT-4 vs Google Bard	*.045*
OpenAI ChatGPT-4 vs Microsoft Bing Chat	*.049*
Google Bard vs Microsoft Bing Chat	.65

^a^Statistically significant values are italicized.

### Qualitative Results

#### Overview

The free-text qualitative comments of the evaluators were reviewed, analyzed, and grouped into key themes (Textbox 1).

**Examples of the evaluators’ comments (exact copies).**

**Scientific correctness and relevance**

- “This is exactly the answer you are looking for” (Microsoft Bing Chat, operative dentistry question).
- “Perfectly correct answer” (Microsoft Bing Chat, operative dentistry question).
- “The answer is correct and it gives further details proving thorough knowledge of the topic” (Google Bard, endodontic question).
- “This answer includes all the findings mentioned in the ESE guidelines and gives additional details regarding causes of RCT failure” (Open AI ChatGPT-4, endodontic question).
- “Additionally, answer No 8 even though not included in the answer key is also correct, so I would have given an additional mark” (Open AI ChatGPT-4, prosthodontic question).
- “It says to remove all carious tissue, that is a mistake (we expected the selective caries removal protocol)” (Open AI ChatGPT-3.5, operative dentistry question).
- “The terminal point for chemo-mechanical preparation and obturation of a given root canal, does not depend on the obturation method applied or material used nor clinician’s experience and preferences!!!!!” (Open AI ChatGPT-3.5, endodontic question).
- “Also does mistakes such as chlorhexidine mouthwash to reduce caries” (Open AI ChatGPT-4, operative dentistry question).
- “This answer is focused on clinical findings without considering the radiographic follow up. Moreover, it mentions some causes of failure which is relevant information but doesn’t answer the question” (Google Bard, endodontic question).
- “The answer is not specifically related to the question” (Google Bard, endodontic question).
- “Half of the answer is correct, but not specific” (Open AI ChatGPT-3.5 and ChatGPT-4, operative dentistry question).
- “Both answers from LLMA and LLMB are correct and similar” (Open AI ChatGPT-3.5 and ChatGPT-4, operative dentistry question).

**Content quality**

- “Well organized answer!” (OpenAI ChatGPT-3.5, operative dentistry question).
- “This is the most comprehensive answer compared to the other 3. Provides regimens and doses” (OpenAI ChatGPT-3.5, oral surgery question).
- “Vague answer” (Microsoft Bing Chat, oral surgery question).
- “Correct answer but not complete” (Microsoft Bing Chat, endodontic question).
- “The answer is incomplete” (Microsoft Bing Chat, endodontic question).
- “Incomplete response, key points omitted” (OpenAI ChatGPT-3.5, OpenAI ChatGPT-4, and Microsoft Bing Chat, oral pathology question).
- “It is on topic but not updated” (Microsoft Bing Chat, endodontic question).
- “This answer is not thorough” (Microsoft Bing Chat, endodontic question).
- “Few points are not mentioned clearly. Other points are not explained well” (OpenAI ChatGPT-4, prosthodontic question).
- “Neutral answer. Two positive and two negative effects. So, no clear direction” (ChatGPT-4, orthodontic question).
- “The answer here is not specific for answering the question” (OpenAI ChatGPT-4, operative dentistry question).
- “It gives me the impression of not understanding the topic in depth” (OpenAI ChatGPT-4 & Google Bard, oral pathology question).
- “I would give some marks but not full marks as the answer is very brief” (Microsoft Bing Chat, prosthodontic question).

**Language**

- “Very good answer, but directed to patients (not dentists)” (Google Bard, oral pathology question).
- “It is not obvious that the answer is given by AI. It could be a dentist with a relatively good knowledge of the literature, although not completely up-to-date” (OpenAI ChatGPT-3.5, endodontic question).
- “Gives the impression that it is a written informal response to an informational question” (Microsoft Bing Chat, endodontic question).
- “The answer is directed to the patient rather than the dentist. This is not what we are looking for in this question” (Google Bard, operative dentistry and oral surgery question).
- “Patients can understand well from this answer” (OpenAI ChatGPT-4, oral surgery question).
- “This is a very similar answer to what many students may have actually answered” (Microsoft Bing Chat, prosthodontic question).

#### Scientific Correctness and Relevance

In general, the LLMs’ responses were scientifically correct and relevant to the questions asked, and sometimes they were even superb. Occasionally, LLMs provided additional relevant content outside the immediate scope of the question, thus enriching the response. Unfortunately, the additional content was not always beneficial: “included information that was not asked” (all LLMs, pediatric dentistry question). Scientifically incorrect, partially correct, or irrelevant answers were also noted. Similar answers from different LLMs were identified. Inability to provide an answer was registered for an LLM on 2 occasions: “I’m unable to help, as I am only a language model and don’t have the ability to process and understand that” (Google Bard, oral surgery question) and “I’m a language model and don’t have the capacity to help with that” (Google Bard, prosthodontic question).

Scientifically incorrect answers were provided by Google Bard and Microsoft Bing for question 2, “what is the recommendation to treat a non-cavitated caries lesion that is limited to enamel and the outer third of dentin, on a proximal surface?” which were graded 1/10 and 2.5/10, respectively. Although evidence appears in the guidelines and consensus statements of international organizations (eg, the FDI and ADA), Google Bard and Microsoft Bing Chat, which claim to have access to the internet, could not retrieve this information. ChatGPT-4 and ChatGPT-3.5 answered the same question correctly, and both scored 9/10.

#### Content Quality (Clarity, Comprehensiveness, and Up-to-Date Knowledge)

The evaluators commented on the quality of the responses, highlighting some positive examples regarding the structure, organization, and clarity of the texts. An example of clear, updated answers was noticed for question 8, “what is the recommended age for a child’s first dental visit?” To this question, all LLMs correctly answered (graded 9/10) that the first visit should take place when the first primary tooth appears and up to 12 months of age, a recommendation that appears in both the American Academy of Pediatric Dentistry and the ADA websites; however, no contradictory information appears on the web, which could possibly confuse the LLMs. The evaluators also noted that some responses were unclear; very brief; very general; outdated; or did not include all the desired, important points. For example, question 5, “What is the material of choice for direct pulp capping (vital pulp therapy)?” for which both older and updated guidelines exist, confused the LLMs, although the updated guidelines were issued well before the knowledge cutoff date of September 2021 for ChatGPT. The only LLM that clearly answered correctly was Google Bard (graded 8.5/10), whereas Microsoft Bing Chat presented the older guidelines as recent guidelines (graded 0.5/10). Contradictory statements within the same answer also appeared and were commented on: “sealants cannot be placed in proximal surfaces and it mentions that before” (Google Bard, operative question).

#### Language

According to the context of the input text (scientifically formatted prompt), the LLMs generated responses in a similar format (scientific language), but not always. We noted language discrepancies, such as “chewing surfaces of the back teeth” instead of “occlusal surfaces of posterior teeth” (Google Bard, operative dentistry question), and the evaluators also noted these incompatibilities. They evaluated the language as being informal sometimes, where the answers seemed as though they were composed by a student or intended for the general public and patients.

References were cited in Microsoft Bing Chat’s responses, although the authors did not specifically ask for them in their queries, apparently because of the recognition of the input text’s formal, scientific language by the LLM, but these references were not always accurate, as either they were either nonexistent or they redirected the reader to an irrelevant document: “I was not able to find the reference mentioned in the answer*”* and *“*after following the link indicated in the answer the following reference was retrieved” (Microsoft Bing Chat, endodontic question).

## Discussion

### Principal Findings and Explanations

Although professional and scientific oral health care organizations strive to embed EBD into dental clinical practice through the development and dissemination of clinical practice guidelines, ongoing challenges such as rapid scientific and technological developments, outdated guidelines, a lack of evidence, and practice workflow obstruct successful implementation [33]. The recent wave of GenAI chatbots, theoretically capable of instantly generating evidence-based responses to scientific queries and thus acting as the dentist’s “chairside personal scientific consultant,” appears to have the potential to be an ideal tool for the successful implementation and enhancement of EBD. To investigate this immersive opportunity, we evaluated 4 LLMs’ responses to queries related to different dental procedures and clinical decision-making processes encountered in routine practice. The responses generated by ChatGPT-4 were provided the highest scores by the evaluators (mean average score 7.2, SD 1.8; range 3.5-10), followed by those generated by ChatGPT-3.5, Google Bard, and Microsoft Bing Chat (mean average score 5.4, SD 3.4; range 0.5-10), and the differences between the first LLM and the others were statistically significant.

ChatGPT-4’s high score can be attributed to its large database, more reliable availability, and extensive training. ChatGPT (and similar LLMs) is a natural language model trained on a vast and diverse amount of data using supervised fine-tuning, reward modeling, and reinforcement learning to generate contextually relevant and humanlike output in response to a text input (prompt, query, and statement) [28,34]. As with any process that requires continuous training to improve and reduce its failures, AI tools require large data sets to train themselves [35], and ChatGPT has been trained for a number of years using such data sets. The first version of ChatGPT, trained on a massive data set of internet-derived text, was launched by OpenAI in June 2018, and a number of updated versions followed until June 2020, when ChatGPT-3, a large and powerful model was released, including 175 billion parameters [13]. Continuous development and refinement of the model’s capabilities resulted in ChatGPT-3.5 in November 2022, followed by the latest model, ChatGPT-4, in March 2023.

The LLMs’ ranking in this study could reflect the differences between them in terms of their architecture, training data, and performance characteristics, which impact their accuracy, relevance, and suitability for different applications or use cases. It should be noted that Google Bard and Microsoft Bing Chat claim to have live access to the internet, whereas the data set knowledge cutoff for ChatGPT is only September 2021.

Although these LLMs are all language models and share similarities, they are based on different neural network architectures: (1) ChatGPT is based on the GPT architecture, a deep-learning technique that involves training the model on massive data before fine-tuning it on specific tasks; (2) Google Bard is based on Google’s LaMDA neural network architecture, designed to allow the model to better understand the context and generate accurate responses; and (3) Microsoft Bing Chat AI is based on a variety of learning models (including GPT-4), depending on the specific task or application. The different network architectures and differences in the amount and diversity of training data result in the LLMs generating different responses to identical questions and having different strengths, weaknesses, capabilities, and limitations overall, whereas similarities also exist. A study by Rudolph et al [36] that compared the same chatbots as those in this study in terms of their use in higher education found the same results, with ChatGPT-4 scoring the best, followed by ChatGPT-3.5 and then Google Bard and Microsoft Bing Chat.

In this study, all LLMs performed relatively well in answering a range of clinically relevant questions (mean average score ranging from 5.4 to 7.2 out of 10). Although ChatGPT-4’s answers appeared superior, we consider this as reflecting the specific conditions of this study, that is, the specific questions asked in a specific manner and at a specific time point. In addition, the evidence deduced from the quality comments can prove to be equally interesting and useful. Overall, the evaluators identified examples of accurate, well-articulated responses, although in most cases, the responses were incomplete, compared with traditional evidence. In several cases, however, the machines were “hallucinating,” with the answers being misleading or wrong, and these answers were presented in an indisputable, expert manner, making them something that could misguide the clinician if they were unfamiliar with the recent developments on the subject.

Undeniably, LLMs possess no factual knowledge of dentistry, medicine, or other sciences [12]; therefore, their errors and inconsistencies could be related to their operation processes. When asked a question, ChatGPT takes in the input text sequence; encodes it into numerical vectors using a process called “tokenization” (ie, breaking the text into words and subwords); passes it through the transformer network, which uses attention mechanisms to weigh the importance of different parts of the input sequence; and generates a corresponding and contextually relevant output sequence [37]. Any mishap in this process will result in an incorrect, an irrelevant, or a confusing response.

Another possible explanation for wrong or inaccurate answers (and their deviation from the established “gold standard”) could be attributed to the fact that the prompts must be very specific for the results to be accurate, as LLMs’ outputs are sensitive to the level of detail in the question; therefore, some questions were probably not phrased accurately enough for the LLMs to correctly perceive them [38]. In addition, in medical and dental AI, deficiencies in the representativeness of the training data sets (different for the different LLMs) may result in inadequate answers [39]. For medical and dental questions, the LLMs need access to specialized knowledge and high-quality and relevant scientific data, which they may not currently have, as they are trained on general text data, possibly not including domain-specific content [13]. In addition, LLMs are unable to understand the complex relationships between medical conditions and treatment options and provide relevant answers [17].

### Comparison With Relevant Literature

Rao et al [22] used a similar research design to evaluate ChatGPT’s capacity for clinical decision support in radiology via the identification of appropriate imaging services for 2 clinical presentations, namely the breast cancer screening and breast pain, and compared ChatGPT’s responses with the American College of Radiology Appropriateness Criteria (apparently used as the “gold standard”). ChatGPT scored high in open-ended questions (average 1.83 out of 2) and was impressively accurate in responding to select all that apply prompts (on average, 88.9% correct responses) for breast cancer screening. ChatGPT displayed more reasoning for open-ended prompts, where it often provided an extensive rationale for recommending the specific imaging modality in accordance with the American College of Radiology Appropriateness Criteria [22].

The evaluators’ qualitative comments were of particular interest, as they reported instances where LLMs included additional content outside the immediate scope of the question or some very brief, very general, and outdated content in their responses. Furthermore, incorrect references were cited, and partially correct, incorrect, confusing, or irrelevant answers were noted, as were 2 “no reply” answers from Google Bard. Such failures and shortcomings of LLMs have also been reported in the relevant recent literature. Abstracts generated by ChatGPT were evaluated as “superficial and vague” [40], and responses to medical questions “were not assumed as fully accurate and authenticated” [13]. In a systematic review on ChatGPT’s applications in health care, Sallam [14] reported incorrect information in one-third of the records studied, inaccurate references in 16.7% of the records, misinformation in 8.3% of the records, and overdetailed content in 8.3% of the records [14].

Fergus et al [15] evaluated ChatGPT-generated responses to chemistry assessment questions and concluded that the quality of the responses varied. For the answers of 10 (62%) out of the 16 questions asked, mostly related to the application and interpretation of knowledge, the evaluators assigned the grade 0, as the answers were incorrect or there was no answer. Interestingly, 1 response was incorrect, although the correct answer could be easily found on the internet [15]. Furthermore, as in our study, the evaluators commented that there were general answers to some questions, omitted key points, and irrelevant additional information.

Patel and Lam [41] described ChatGPT’s ability to produce a patient’s discharge summary and reported that the LLM added extra information to the summary that was not included in the input prompt. Similarly, in a separate study testing ChatGPT’s ability to simplify radiology reports, key medical findings were reported as missing [42]. Vaishya et al [13] interacted with ChatGPT and identified incorrect information in multiple places, factual mistakes in responses to medical questions, and different responses to the same questions with a lot of general information. LLMs can generate entirely wrong or inaccurate, biased, or even harmful responses; fabricate information; and present the fabricated information as real (“hallucinations”); all these issues raise major concerns in health care practice, particularly when reliable evidence is sought to inform clinical practice and the decision-making process [12,20,22,28,30].

Mago and Sharma [38] asked ChatGPT-3 80 questions on oral and maxillofacial radiology, related to anatomical landmarks, oral and maxillofacial pathologies, and the radiographic features of pathologies, and the answers were evaluated by a dentomaxillofacial radiologist. They concluded that ChatGPT-3 was overall efficient and can be used as an adjunct when an oral radiologist requires additional information on pathologies; however, it cannot be the main reference source. ChatGPT-3 does not provide the necessary details, and the data possess a risk of infodemics and the possibility of medical errors [38].

### Clinical Practice: Applications, Challenges, Limitations, and Future Directions of LLMs

Although dental professionals are dedicated to providing the best care for their patients, several challenges exist, resulting in clinicians not yet being fully aligned with the concept of EBD, which would facilitate clinical decision-making and improve treatment outcomes in oral health care [43]. User-friendly and fast-growing LLMs may have the potential to become valuable tools in office practice and enhance diagnostic accuracy, clinical decision-making, treatment planning, patient communication, and oral health literacy [14,20]. Current research on LLMs mainly explores the ChatGPT tool and is limited to education, research, scientific writing, and patient information, whereas clinical perspectives have a limited evidence level.

In respect to patients, patient-centered oral health care could be further promoted, with patients having access to information regarding their health status, thus empowering them to make informed decisions. For example, Balel [44] concluded in his study that ChatGPT has significant potential as a tool for patient information in oral and maxillofacial surgery. However, patients should correctly understand and interpret the information they obtain from the chatbot, and health care professionals should verify its accuracy [44]. Patients can describe their symptoms, ask questions, and receive explanations, thus better understanding their treatment options and diagnoses; treatment plans may be tailored to the unique needs of each patient, improving the patient-professional relationship [45]. However, patients’ easy and instantaneous access to medical information (or misinformation) may challenge professionals while confronting their opinions and demands.

ChatGPT can offer personalized oral hygiene advice to help patients maintain good oral health, prevent common dental problems, and increase their oral health literacy and awareness. It can also provide postprocedure instructions and medication reminders, as well as offer relaxation techniques and coping strategies to patients with stress [46].

In respect to clinicians and medical or dental professionals, LLMs, such as ChatGPT, could play a role in diagnosis and treatment planning by analyzing patients’ symptoms, history, and clinical signs, thus serving as a clinical decision support system (eg, for oral diseases and rare pathologies) [47].

In the field of oral and maxillofacial surgery, LLMs could transform perioperative care for patients and surgeons. When asked about relevant potential applications, GPT-4 included patient interaction, surgical planning and decision-making, assistance in clinical documentation (eg, writing of discharge letters), remote consultations, psychological support, and protocol and guideline reminders [48].

Among specialist professionals, ChatGPT can serve as a platform for knowledge sharing and collaboration by facilitating discussions on complex cases; enabling professionals to consider diagnostic and treatment possibilities outside their routine practices, the sharing of research findings, and brainstorming; and providing a virtual space for exchanging expertise and best practices [45,49].

An important issue is that LLMs do not provide the sources of the information they use, and this is a major problem, as verification is difficult, if not impossible, albeit necessary. This, in combination with the fact that LLMs were created by commercial companies and without any governmental or other type of legislation or control so far, may lead to information platforms with unknown goals that are potentially against the benefit of societies, public health, and safe and effective evidence-based treatment.

Transparency (the capacity to attribute factual information to its source and openness of the sources), as well as all ethical and technical guidelines regulating the use of these machines and controlling their application, should be ruled by solid legislation, which should be developed as soon as possible and serve, among other roles, as a scientific gatekeeper for evidence-based health care. In the margins of the EU-US Trade and Technology Council, a stakeholder panel named “Perspectives on Large AI Models” brought together EU and US representatives, including the US Secretary of State Anthony Blinken; European Commission Executive Vice President Margrethe Vestager; and stakeholders representing industry, academia, and civil society [50]. The need to prepare to address the broader effects of AI on economies and societies and to regulate AI systems directly to ensure that AI benefits society has also been stressed by the representatives of international organizations such as the International Monetary Fund [51]. The International Organization for Standardization (ISO) and the International Electrotechnical Commission (IEC) have already established concepts, terminology, and frameworks related to AI and machine learning [52,53]. Hopefully, a solid and detailed regulatory foundation will soon exist for AI technology [54].

The inherent limitations and weaknesses of LLMs reported in this study, in line with the recent literature, include a lack of reliability (possible inaccurate, irrelevant, or inconsistent responses) and transparency (inability to attribute factual information to its source), possible outdated content, limited database, inability to search the internet, and ethical and societal concerns [12,14,22]. These shortcomings currently curtail the use of LLMs as health care assistance tools, for which the LLMs should be trained with high-quality, continuously updated, and domain-specific data sets and thus become up-to-date, reliable, consistent, and unbiased [12-14]. Before their implementation in evidence-based dental practice, LLMs should be clinically validated, and evidence demonstrating their clinical utility, efficacy, and applicability should be presented [1,14,20,55]. Furthermore, the sources of information should be provided, at least upon request, so that the dentist can evaluate the information, add to it from sources not referenced, and apply critical thinking to it. Meanwhile, oral health care providers need to learn how to improve the queries they ask LLMs so that the latter will produce more relevant replies [28,34].

The future of GenAI LLMs will likely involve ongoing development and performance improvements, for example, through the expansion of their training data and refinement of their algorithms, which would help improve their performance and enhance their ability to generate more complex responses, such as those exhibiting reasoning or a deep understanding of context. A crucial factor for the future applications of LLMs in dentistry is training LLMs with dentistry-specific knowledge, such as teaching material from different sources and patient records and displaying different patterns and terminology, resulting in enhanced accuracy and relevance. Continuous training through machine learning and fine-tuning will update the models’ content to include recent medical developments and knowledge [45]. In addition, the integration of ChatGPT and similar models into scientific databases, such as Web of Science, PubMed, and Scopus, would improve the quality and accuracy of responses to scientific questions; we propose that this new version be named ChatGPT-Academic [44]. Incorporating virtual and augmented reality into the LLMs will fundamentally alter diagnosis and treatment planning [45]. Multimodal LLMs combining various types of input data, such as radiographs; biopsy microscopy images; text; audio input, such as patients’ narratives of history or symptoms; and video, could lead to accurate diagnoses, as well as other applications [56,57]. Already, the new GPT-4 version accepts images (documents with photographs, diagrams, and screenshots) as input queries [58].

On the basis of the aforementioned information, dentists still need to be well educated and as updated as possible through all means of traditional evidence-based education. This would allow them to apply critical thinking to the information provided by LLMs, so it may be used in a positive way. Otherwise, clinicians may easily be misguided. Currently, irrespective of the knowledge data set or training, LLMs do not seem to be able to replace unique human intellectual abilities. Any evaluation and use of this technology should be carried out with skepticism and a high level of critical thinking. We propose that health professionals and academicians should be cautious when using ChatGPT and similar models, compare them with reputable sources, and consider them as a supplement to their clinical knowledge and experience [44,49]. Clinicians must be very alert and apply all means of evaluation and criticism to the information provided before such tools are established as support for clinical decision-making and EBD. This is in line with what ChatGPT admitted: “while I can generate text that is related to scientific evidence and clinical decision-making, it is important to note that I am not a substitute for professional medical advice, diagnosis, or treatment.” [59].

### Strengths and Limitations

This study has several strengths, the most important of which is that, to our knowledge, this is the first research study to show that LLMs are related to EBD, which seems to be an excellent combination, considering the clinical practice environment and the capabilities of LLMs. Moreover, 4 LLMs were examined simultaneously, which is a rare methodology, as almost all studies retrieved investigated only 1 model, usually ChatGPT, as it was the first to appear for public use and the most prominent one. A third strength is that apart from the quantitative results, the study presents qualitative results (the evaluators’ quality comments), which offer detailed insights into the LLMs’ performance and highlight some of the LLMs’ limitations.

A limitation of our study could be that the questions were asked only once, with no follow-up questions or requests for additional clarifications, which could have produced more relevant and less inaccurate answers. Consequently, the ability of the LLMs to generate evidence-based responses could have possibly been underestimated. Because it has been reported that ChatGPT may generate different responses to the same prompt if asked multiple times (or to a slightly modified prompt), by different users [15,40], or at different times [13], we chose not to complicate the research design by introducing additional parameters. In addition, limiting interactions to single queries allowed for a more focused evaluation of the LLMs’ ability to provide concise and relevant responses to queries without the need for reprompting, meaning that the process could be once-off and not time consuming, thus mirroring real-world clinical practice.

The concept of “gold standards” could also be considered a limitation, as guidelines and organizations’ recommendations may differ within countries or continents and may not be universally accepted. We tried to address this by choosing consensus and high-quality “gold standards,” which still may not be universally applicable. Finally, it should be noted that the answers reflect the LLMs’ performance at the time of research and that their performance may change over time, which is an inherent limitation of studies involving technological developments.

### Conclusions

The implementation of LLMs such as ChatGPT in evidence-based clinical practice looks promising; however, extensive research and clinical validation as well as model improvements are needed to address their inherent weaknesses. Until GenAI and LLMs reach their full potential, health care professionals should judiciously and critically use them to inform their clinical practice.

The 4 LLMs evaluated herein in terms of their responses to clinically relevant questions performed rather well, with ChatGPT-4 exhibiting the statistically significantly highest performance and Microsoft Bing Chat exhibiting the lowest. Irrespective of the LLMs’ ranking, the evaluators identified similar advantages, weaknesses, and limitations, including occasional inaccuracies, errors, outdated or overgeneral content, and contradictory statements. Although the widespread use of LLMs offers an opportunity to reinforce the implementation of EBD, the current limitations suggest that imprudent use could result in biased or potentially harmful health care decisions.

## Appendix

Multimedia Appendix 1 Clinical dentistry–related questions asked to the large language models.

Multimedia Appendix 2 The assessment rubric used to evaluate the questions.

Multimedia Appendix 3 The answers of the large language models (LLMs) to the questions asked and references to the scientific evidence. A summary of the information that the LLMs’ answers were graded against is provided for the convenience of the reader.

## Abbreviations

ACR: American College of Radiology
ADA: American Dental Association
AI: artificial intelligence
EBD: evidence-based dentistry
FDI: Federation Dentaire Internationale
GenAI: generative artificial intelligence
GPT: generative pretrained transformer
ICC: intraclass correlation coefficient
IEC: International Electrotechnical Commission
ISO: International Organization for Standardization
LaMDA: Language Model for Dialogue Applications
LLM: large language model
PaLM: Pathways Language Model

## Data Availability

Any data not appearing in this paper are available from the corresponding author upon reasonable request.
