# Supplementary material for: Evaluation of the Performance of Generative AI Large Language Models ChatGPT, Google Bard, and Microsoft Bing Chat in Supporting Evidence-Based Dentistry: Comparative Mixed Methods Study
Source: J Med Internet Res. 2023 Dec 28;25:e51580. doi: 10.2196/51580 (PMC10784979; doi:10.2196/51580)
Supplement: Multimedia Appendix 1 [file jmir_v25i1e51580_app1.docx]

**Clinical dentistry-related questions queried to the LLMs:**

1. According to the American Heart Association, what is the antibiotic regime to be prescribed to a high-risk patient for infective endocarditis?

2. What is the recommendation to treat a non cavitated caries lesion that is limited to enamel and the outer third of dentin, on a proximal surface?

3. Why is it recommended to phase down dental amalgam?

4. How do you approach the removal of carious tissue in dentin cavities and symptomless teeth?

5. What is the material of choice for direct pulp capping (vital pulp therapy)?

6. What radiographs and how often should be prescribed for an adult recall patient with clinical caries or at increased risk for caries? What radiographs and how often should be prescribed for an adult recall patient with no clinical caries and with no increased risk for caries?

7. What are the indications to remove wisdom teeth?

8. What is the recommended age for a child's first dental visit?

9. What is recommended to arrest a cavitated caries lesion in primary teeth?

10. What tooth is the most bilaterally symmetrical in the human permanent dentition?

11. What is the ideal apical terminus of shaping and obturation of the root canal during endodontic treatment?

12. In surgical extrusion and intentional replantation, what is the maximum duration of extra alveolar manipulation to prevent damage of the periodontal ligament?

13. What are the indications and contraindications for administration of systemic antibiotics in endodontics?

14. Which findings indicate an unfavorable outcome after endodontic treatment?

15. What are the recommended radiation protection measures to be taken in the dental office for the protection of patients, doctors and personnel from unnecessary radiation exposure?

16. What are the objectives of surveying the diagnostic cast during partial denture construction?

17. What are the precautions that should be taken during light curing in dentistry?

18. What are the distinguishing features of solitary vs. proliferative leukoplakia of the oral mucosa?

19. Is early orthodontic treatment in two phases for children with prominent upper teeth, more beneficial compared to treatment that is provided in one phase in adolescence?

20. Does orthodontic treatment affect the airway function?
