# Supplementary material for: Evaluation of the Performance of Generative AI Large Language Models ChatGPT, Google Bard, and Microsoft Bing Chat in Supporting Evidence-Based Dentistry: Comparative Mixed Methods Study
Source: J Med Internet Res. 2023 Dec 28;25:e51580. doi: 10.2196/51580 (PMC10784979; doi:10.2196/51580)
Supplement: Multimedia Appendix 2 [file jmir_v25i1e51580_app2.docx]

**ASSESSMENT RUBRIC**

| **Criteria** | **0** | **1-2** | **3-4** | **5-7** | **8-9** | **10** | **Rating** |
| --- | --- | --- | --- | --- | --- | --- | --- |
| **Comprehensiveness**  **[25%]** | Response is not at all comprehensive | Very few key points are included | A basic level of key points is included | Many key points are included, but still some key information is missing | Almost all key points are included | All key points are included |  |
| **Scientific accuracy**  **[25%]** | Response completely inaccurate | Response mostly inaccurate | Response presents a basic level of accuracy | Response exhibits a good level of scientific accuracy, but some key points are not presented accurately | Almost all key points are presented accurately | All key points are presented accurately |  |
| **Clarity**  **[25%]** | Response not clear at all, leaving the assessor very confused | Response obscures most key points | Response obscures many key points | Response is mostly clear, but some key points are not | Almost all key points are presented clearly | All key points are presented clearly |  |
| **Relevance**  **[25%]** | Response not relevant at all | Many irrelevant points included | Some irrelevant points included | Most points included are relevant | Almost all points included are relevant | All points included are relevant |  |
| **Total score [rounded to the next integer]** | | | | | | |  |
