# Supplementary material for: Evaluation of the Performance of Generative AI Large Language Models ChatGPT, Google Bard, and Microsoft Bing Chat in Supporting Evidence-Based Dentistry: Comparative Mixed Methods Study
Source: J Med Internet Res. 2023 Dec 28;25:e51580. doi: 10.2196/51580 (PMC10784979; doi:10.2196/51580)
Supplement: Multimedia Appendix 3 [file jmir_v25i1e51580_app3.docx]

| **Questions/discipline** | **ChatGPT GPT-3.5** | **ChatGPT GPT-4** | **Google Bard** | **Microsoft Bing Chat** | **Evidence (literature) and summary** |
| --- | --- | --- | --- | --- | --- |
| 1. According to the American Heart Association, what is the antibiotic regimen to be prescribed to a high-risk patient for infective endocarditis?  Discipline: General Dentistry/Oral Surgery/Oral Pathology | The American Heart Association (AHA) revised its guidelines for the prevention of infective endocarditis in 2007. According to these guidelines, antibiotics are recommended for high-risk patients undergoing certain dental procedures or surgical procedures involving infected tissue. The recommended regimen for high-risk patients is:   1. Amoxicillin 2 grams orally, 1 hour before the procedure, OR 2. Ampicillin 2 grams intravenously or intramuscularly, 30 minutes before the procedure, OR 3. Cefazolin 1 gram intravenously or intramuscularly, 30 minutes before the procedure, OR 4. Clindamycin 600 mg orally, 1 hour before the procedure, OR 5. Azithromycin or clarithromycin 500 mg orally, 1 hour before the procedure (for patients who are allergic to penicillin and unable to tolerate other antibiotics).   It's important to note that these recommendations are for patients who are at high risk for infective endocarditis. Patients who are not at high risk may not require antibiotic prophylaxis. Additionally, individual patient factors and medical history should always be considered when making decisions about antibiotic prophylaxis. It is recommended to consult with a healthcare professional to determine the best course of action for each patient. | I am not a doctor, but I can provide you with some information about infective endocarditis prophylaxis based on the American Heart Association's (AHA) guidelines as of my last knowledge update in September 2021. Please note that guidelines may have changed since then, and it is important to consult with a healthcare professional for the most current recommendations.  In 2021, the AHA recommended antibiotic prophylaxis for specific high-risk patients undergoing certain dental, respiratory, or invasive procedures. The antibiotic regimen for dental procedures in patients with high risk of infective endocarditis was typically a single dose of amoxicillin (2 grams) given orally one hour before the procedure. For patients allergic to penicillin, alternative antibiotics like clindamycin, cephalexin, or azithromycin could be used.  It's important to note that the AHA guidelines and recommendations may change over time, and the specific antibiotic regimen prescribed could vary depending on the patient's medical history and other factors. Always consult a healthcare professional for the most accurate and up-to-date information. | I'm unable to help, as I am only a language model and don't have the ability to process and understand that.  Second time the question was asked:  I'm not programmed to assist with that. | [According to the American Heart Association, people with the highest risk for poor outcomes from infective endocarditis may be prescribed antibiotics (IE prophylaxis) prior to certain dental procedures to reduce their risk of developing it1](https://www.heart.org/en/health-topics/infective-endocarditis). [The guidelines for antibiotic prophylaxis for infective endocarditis have changed over time and are now more restrictive than in the past2](https://www.ahajournals.org/doi/10.1161/CIRCULATIONAHA.119.041085). [The current guidelines recommend that only patients at highest risk of developing infective endocarditis should receive prophylaxis3](https://www.ahajournals.org/doi/10.1161/CIR.0000000000000296).  I’m sorry but I couldn’t find a specific antibiotic regime for high-risk patients for infective endocarditis. If you have any other questions, please let me know.  Learn more:  [1. heart.org](https://www.heart.org/en/health-topics/infective-endocarditis)[2. ahajournals.org](https://www.ahajournals.org/doi/10.1161/CIRCULATIONAHA.119.041085)[3. ahajournals.org](https://www.ahajournals.org/doi/10.1161/CIR.0000000000000296) | Wilson WR, Gewitz M, Lockhart PB, Bolger AF, DeSimone DC, Kazi DS, Couper DJ, Beaton A, Kilmartin C, Miro JM, Sable C, Jackson MA, Baddour LM; American Heart Association Young Hearts Rheumatic Fever, Endocarditis and Kawasaki Disease Committee of the Council on Lifelong Congenital Heart Disease and Heart Health in the Young; Council on Cardiovascular and Stroke Nursing; and the Council on Quality of Care and Outcomes Research. Prevention of Viridans Group Streptococcal Infective Endocarditis: A Scientific Statement From the American Heart Association. Circulation. 2021 May 18;143(20):e963-e978. doi: 10.1161/CIR.0000000000000969. Epub 2021 Apr 15. Erratum in: Circulation. 2021 Aug 31;144(9):e192. Erratum in: Circulation. 2022 Apr 26;145(17):e868. PMID: 33853363.  Summary of evidence:  2gr amoxicillin 1 hr before the procedure.   \| Unable to take oral medication \| Ampicillin **OR** \| 2 g IM or IV \| 50 mg/kg IM or IV \| \| --- \| --- \| --- \| --- \| \| Cefazolin or ceftriaxone \| 1 g IM or IV \| 50 mg/kg IM or IV \|  \| Azithromycin or clarithromycin \| 500 mg \| 15 mg/kg \| \| --- \| --- \| --- \|   Doxycycline  100 mg  <45 kg, 2.2 mg/kg >45 kg, 100 mg  Allergic to penicillin or ampicillin and unable to take oral medication  Cefazolin or ceftriaxone†  1 g IM or IV  50 mg/kg IM or IV  Clindamycin is no longer recommended for antibiotic prophylaxis for a dental procedure. IM indicates intramuscular; and IV, intravenous. |
| 2. What is the recommendation to treat a non-cavitated caries lesion that is limited to enamel and the outer third of dentin, on a proximal surface?  Discipline: Operative Dentistry | The recommended treatment for a non-cavitated caries lesion that is limited to enamel and the outer third of dentin on a proximal surface is to arrest the lesion through non-surgical management. This involves the following steps:  Oral hygiene instructions: Provide oral hygiene instructions and reinforce the importance of maintaining good oral hygiene practices, including brushing twice a day with a fluoride toothpaste, flossing daily, and limiting sugary foods and drinks.  Fluoride therapy: Apply fluoride varnish or prescribe fluoride mouthwash or gel to increase the remineralization potential of the affected tooth structure.  Dietary counseling: Provide dietary counseling to reduce the frequency and amount of fermentable carbohydrates, which are the primary substrate for cariogenic bacteria.  Monitoring: Regularly monitor the lesion's progress through clinical examination and radiographic assessment to ensure that the lesion is stable or arrested.  Sealant placement: Consider placing a dental sealant over the affected area to help prevent further demineralization and promote remineralization.  Restoration: If the lesion progresses or shows signs of cavitation, consider restoring the tooth with a minimally invasive restoration such as a composite resin restoration.  It's essential to remember that early detection and non-surgical management of non-cavitated caries lesions can prevent the need for more invasive and costly treatment in the future. | I am not a dentist, but I can provide some general information about treating non-cavitated caries lesions. It's important to consult with a dental professional for personalized advice.  For a non-cavitated caries lesion limited to the enamel and the outer third of dentin on a proximal surface, a conservative treatment approach is usually recommended. This may include:   1. Oral hygiene instructions: Emphasize the importance of proper oral hygiene, including regular toothbrushing with a fluoride toothpaste, flossing, and using interdental brushes to clean proximal surfaces. 2. Fluoride application: Topical fluoride applications, such as fluoride varnish or gel, can help remineralize and strengthen the tooth structure, preventing further progression of the caries lesion. 3. Dietary counseling: Limit the intake of fermentable carbohydrates and sugary foods, and promote a balanced diet to maintain a healthy oral environment. 4. Monitoring: Regular dental check-ups are essential to monitor the lesion's progression and determine if further intervention is required. 5. Sealants or resin infiltration: In some cases, dental professionals may recommend using sealants or resin infiltration to prevent the caries lesion from progressing. 6. Antimicrobial agents: The use of antimicrobial agents, such as chlorhexidine mouthwash, may be recommended to reduce bacterial plaque and control caries risk. 7. It is essential to consult with a dental professional to evaluate your specific situation and determine the best course of action. | The recommendation for treating a non-cavitated caries lesion that is limited to enamel and the outer third of dentin, on a proximal surface, is to place a dental sealant. A dental sealant is a thin, plastic coating that is applied to the chewing surfaces of the back teeth. It helps to prevent cavities by filling in the pits and fissures where food and bacteria can get stuck.  Sealants are a safe and effective way to prevent cavities, especially in children and adolescents. They are also relatively inexpensive and can be applied in a single dental visit.  If you have a non-cavitated caries lesion, talk to your dentist about whether a sealant is right for you. | According to a study published in the International Dental Journal, all initial caries lesions should be treated by the use of topical fluoride and monitored for progression. [Further options are the use of fissure sealant in occlusal lesions and may include resin infiltration in proximal lesions into the outer third of the dentine 1](https://onlinelibrary.wiley.com/doi/10.1111/idj.12551)[2](https://onlinelibrary.wiley.com/doi/full/10.1111/idj.12551)[3](https://www.fdiworlddental.org/carious-lesions-and-first-restorative-treatment).  Learn more:  [1. onlinelibrary.wiley.com](https://onlinelibrary.wiley.com/doi/10.1111/idj.12551)[2. onlinelibrary.wiley.com](https://onlinelibrary.wiley.com/doi/full/10.1111/idj.12551)[3. fdiworlddental.org](https://www.fdiworlddental.org/carious-lesions-and-first-restorative-treatment) | Enzo Bondioni. Carious lesions and first restorative treatment: Adopted by the General Assembly: September 2019, San Francisco, United States of America. Int Dent J. 2020 Feb;70(1):5-6. doi: 10.1111/idj.12551. PMID: 31985817; PMCID: PMC9379153.  Pitts N, Zero D. *White Paper on Dental Caries Prevention and Management. A summary of the current evidence and the key issues in controlling this preventable disease*, 2016. Available from [https://www.fdiworlddental.org/sites/default/files/media/documents/2016-...](https://www.fdiworlddental.org/sites/default/files/media/documents/2016-fdi_cpp-white_paper.pdf).  Schwendicke F, Splieth C, Breschi L, Banerjee A, Fontana M, Paris S, Burrow MF, Crombie F, Page LF, Gatón-Hernández P, Giacaman R, Gugnani N, Hickel R, Jordan RA, Leal S, Lo E, Tassery H, Thomson WM, Manton DJ, 2019. *When to intervene in the caries process? An expert Delphi consensus statement*. Clinical Oral Investigations. doi: 10.1007/s00784-019-03058-w.  ICCMS Guide for Practitioners and Educators  <https://www.iccms-web.com/uploads/asset/59284654c0a6f822230100.pdf>  Slayton RL, Urquhart O, Araujo MWB, Fontana M, Guzmán-Armstrong S, Nascimento MM, Nový BB, Tinanoff N, Weyant RJ, Wolff MS, Young DA, Zero DT, Tampi MP, Pilcher L, Banfield L, Carrasco-Labra A. Evidence-based clinical practice guideline on nonrestorative treatments for carious lesions: A report from the American Dental Association. J Am Dent Assoc. 2018 Oct;149(10):837-849.e19. doi: 10.1016/j.adaj.2018.07.002. PMID: 30261951.  Summary of evidence: Prevention, oral hygiene instructions, diet modification, Fluoride treatment, recalls, Bitewing x-rays |
| 3. Why is it recommended to phase down dental amalgam?  Discipline: Operative Dentistry | The phasing down of dental amalgam is recommended by various organizations and governments worldwide, including the World Health Organization (WHO), due to concerns over the potential environmental impact and health risks associated with the mercury content in dental amalgam.  Dental amalgam is a mixture of metals that has been used for fillings for over 150 years. It typically contains approximately 50% mercury, along with other metals such as silver, copper, and tin. While dental amalgam is considered safe and effective for most people, the release of mercury vapor during the placement and removal of fillings can pose health risks to patients, dentists, and the environment.  Mercury is a toxic substance that can have harmful effects on the nervous system, kidneys, and immune system. Exposure to high levels of mercury vapor can cause symptoms such as tremors, memory loss, and difficulty concentrating. Additionally, when dental amalgam waste is not properly disposed of, it can contribute to environmental mercury pollution, which can harm wildlife and ecosystems.  For these reasons, many countries and organizations are advocating for a phasing down of dental amalgam use and promoting alternative dental materials, such as composite resin fillings, which are mercury-free and considered safer for both patients and the environment. | Phasing down dental amalgam is recommended for several reasons, primarily due to concerns about its environmental impact and potential health risks. Dental amalgam is a material used for filling cavities in teeth, and it is composed of a mixture of metals, including mercury, silver, tin, and copper. Although it has been used for more than 150 years in dentistry, its use has become more controversial over time.   1. Environmental concerns: Dental amalgam contains mercury, which is a toxic and persistent pollutant. When dental amalgam waste enters the environment, it can contaminate air, water, and soil, posing risks to both wildlife and human populations. Mercury pollution can enter the food chain and bioaccumulate, leading to long-term ecological consequences. 2. Health concerns: There are concerns about the potential health risks of dental amalgam, particularly due to mercury's toxicity. Low levels of mercury vapor can be released from amalgam fillings during everyday activities like chewing and brushing. While the scientific consensus is that the levels of mercury released from dental amalgam fillings are generally considered safe for most people, there are concerns for vulnerable populations, such as pregnant women, young children, and individuals with pre-existing health conditions or impaired kidney function. 3. Alternative materials: Advances in dental materials and technology have led to the development of alternative filling materials, such as composite resins, glass ionomer cement, and ceramic fillings. These alternatives are more biocompatible, less toxic, and in many cases, more aesthetically pleasing compared to dental amalgam. 4. International agreements: The Minamata Convention on Mercury, a global treaty adopted in 2013, aims to protect human health and the environment from anthropogenic emissions and releases of mercury and mercury compounds. As part of this agreement, many countries have committed to phasing down the use of dental amalgam to reduce mercury pollution.   While phasing down dental amalgam is recommended for these reasons, it's essential to consult with dental professionals when making decisions about dental materials and treatments, as each patient's needs and circumstances may vary. | There are a number of reasons why it is recommended to phase down the use of dental amalgam.  Mercury is a neurotoxin. Mercury is a heavy metal that can be harmful to the nervous system. It can be released into the body when dental amalgam is drilled out or when it is broken down over time.  Mercury can be harmful to the environment. Mercury can be released into the environment when dental amalgam is disposed of improperly. It can then be absorbed by plants and animals, and can eventually make its way back to humans through the food chain.  There are safe and effective alternatives to dental amalgam. There are a number of safe and effective alternatives to dental amalgam, such as composite resins and glass ionomers. These materials are just as effective as dental amalgam at filling cavities, and they do not contain mercury.  The Minamata Convention on Mercury is an international treaty that was adopted in 2013. The goal of the convention is to protect human health and the environment from the adverse effects of mercury. The convention includes a provision for phasing down the use of dental amalgam.  As of 2023, more than 130 countries have ratified the Minamata Convention on Mercury. These countries are working to phase down the use of dental amalgam in a way that protects human health and the environment.  If you are considering getting a dental filling, it is important to talk to your dentist about the risks and benefits of dental amalgam. There are safe and effective alternatives to dental amalgam available, and your dentist can help you choose the best option for your needs. | [Dental amalgam is a mixture of metals that has been used for dental fillings for over 150 years1](https://www.bda.org/amalgam). [However, dental amalgam contains mercury which can be harmful to the environment and human health1](https://www.bda.org/amalgam)[2](https://www.nature.com/articles/sj.bdj.2013.737.pdf). [The Minamata Convention on Mercury, which was adopted in 2013, advocates for a phase-down of the use of dental amalgam1](https://www.bda.org/amalgam)[2](https://www.nature.com/articles/sj.bdj.2013.737.pdf). [The phase-down is in line with the domestic circumstances of each country and in tandem with recommendations for prevention programs and increased research into alternative materials1](https://www.bda.org/amalgam). [The phased reduction of the use of dental amalgam is well supported by an increasing focus on caries prevention and research, and development of new cost-effective dental restorative materials with good quality, safety, longevity, adhesive properties, and that are environmentally friendly3](https://fdiworlddental.org/amalgam-part-2-safe-use-and-phase-down-dental-amalgam).  I hope this helps! Let me know if you have any other questions.  Learn more:  [1. bda.org](https://www.bda.org/amalgam)[2. nature.com](https://www.nature.com/articles/sj.bdj.2013.737.pdf)[3. fdiworlddental.org](https://fdiworlddental.org/amalgam-part-2-safe-use-and-phase-down-dental-amalgam)[4. who.int](https://www.who.int/news-room/events/detail/2022/03/11/default-calendar/country-progress-and-way-forward-in-phasing-down-the-use-of-dental-amalgam) | <https://www.fdiworlddental.org/minamata-convention-mercury>  <https://eur-lex.europa.eu/legal-content/EN/ALL/?uri=CELEX%3A32017R0852>  Summary of evidence:  Mercury is a very toxic substance.The use of mercury in production processes should be phased out and, to that end, incentives should be provided for research into alternative substances to mercury with characteristics that are innocuous, or in any case, less dangerous for the environment and for health.  Dental amalgam is the largest use of mercury in the European Union and a significant source of pollution of the environment. The use of dental amalgam should therefore be phased down in accordance with the Convention and with national plans based notably upon the measures listed in Annex A, Part II, to the Convention. The Commission should assess and report on the feasibility of a phase out of the use of dental amalgam in the long term, and preferably by 2030, taking into account national plans required by this Regulation and whilst fully respecting Member States' competence for the organisation and delivery of health services and medical care. Furthermore, particular measures of preventive health protection should be taken for vulnerable members of the population, such as children and pregnant and breastfeeding women.  Hence, the use of mercury in bulk form by practitioners should be prohibited. Amalgam capsules such as described in European standards [EN ISO 13897:2004 and EN ISO 24234:2015] are suitable for use by dentists. Furthermore, minimum retention efficiency of amalgam separators should be set. Compliance of amalgam separators should be based on relevant standards, such as European standard EN ISO 11143:2008.    Why phase down and not phase out: Related to the phase-down of dental amalgam, while much progress has been made with alternative restorative materials, they are not practical for all clinical situations or in settings that lack a reliable source of electricity or other necessary equipment. Thus, it is important to preserve the availability of dental amalgam as a restorative option until investments in research can deliver an alternative restorative material that addresses these current shortcomings. |
| 4. How do you approach the removal of carious tissue in dentin cavities and symptomless teeth?  Discipline: Operative Dentistry | As an AI language model, I don't have the ability to diagnose or provide specific treatment plans for individual patients. However, I can provide some general information on the removal of carious tissue in dentin cavities and symptomless teeth.  In general, the removal of carious tissue in dentin cavities and symptomless teeth is typically accomplished using a dental drill or other mechanical instruments. The goal is to remove all of the decayed or infected tissue from the affected tooth, while preserving as much healthy tissue as possible.  The amount of carious tissue that needs to be removed will depend on the severity of the decay and the extent of the damage to the tooth. In some cases, it may be possible to remove only a small amount of tissue, while in others, more extensive removal may be necessary.  Once the carious tissue has been removed, the dentist will typically fill the cavity with a dental filling material, such as composite resin or amalgam. The filling will help to restore the shape and function of the tooth, while also preventing further decay from developing.  It's important to note that the removal of carious tissue is a critical step in preventing the progression of tooth decay and the development of more serious dental problems. If you suspect that you may have a cavity or other dental issue, it's important to seek professional dental care as soon as possible. | I am not a dentist, but I can provide some general information on the topic. When approaching the removal of carious tissue in dentin cavities and symptomless teeth, dentists typically follow a conservative approach to preserve as much healthy tooth structure as possible. Here are some steps that might be involved in the process:   1. Diagnosis: First, dentists need to accurately diagnose the presence and extent of carious lesions in the teeth. This may involve visual examination, tactile exploration, and radiographic imaging. 2. Anesthesia: Depending on the depth and location of the cavity, local anesthesia might be administered to numb the area and ensure patient comfort during the procedure. 3. Isolation: To maintain a clean and dry working field, dentists may use a rubber dam or other isolation methods. This helps to prevent contamination and improve the effectiveness of the treatment. 4. Caries removal: Dentists use various instruments, such as dental handpieces, burs, or excavators, to carefully remove the decayed dentin. They might employ a stepwise approach, initially removing the outer infected dentin and leaving the affected dentin near the pulp. 5. Assessment: Dentists assess the progress of the caries removal periodically. They use visual, tactile, or other diagnostic methods to ensure they have removed enough carious tissue without unnecessarily removing healthy tooth structure. 6. Pulp protection: If the cavity is close to the pulp, dentists may place a protective liner or base, such as calcium hydroxide or a glass ionomer, to promote dentin repair and prevent pulp exposure. 7. Restoration: After caries removal, the cavity is filled with a suitable dental restorative material, such as composite resin, amalgam, or glass ionomer cement, to restore the tooth's function and appearance. 8. Follow-up: Regular dental check-ups are important to monitor the treated tooth and ensure the success of the restoration.   It is important to consult with a licensed dental professional for personalized advice and treatment. | The approach to removal of carious tissue in dentin cavities and symptomless teeth is called selective caries removal. It is a minimally invasive approach that aims to remove only the carious tissue that is necessary to prevent further decay, while preserving as much tooth structure as possible.  The steps involved in selective caries removal are as follows:  The dentist will assess the extent of the carious lesion and determine the appropriate level of removal.  The dentist will use hand instruments or rotary instruments to remove the carious tissue.  The dentist will etch the exposed dentin with phosphoric acid.  The dentist will place a restorative material, such as amalgam, composite resin, or glass ionomer cement, to fill the cavity.  Selective caries removal has several advantages over traditional methods of cavity preparation, such as:  It is less invasive, which reduces the risk of pulp exposure and tooth fracture.  It preserves more tooth structure, which can improve the strength and longevity of the tooth.  It is less painful, and it may require fewer dental visits.  Selective caries removal is a safe and effective way to treat carious lesions in symptomless teeth. It is a good option for patients who want to avoid the more invasive traditional methods of cavity preparation.  Here are some additional tips for patients who are considering selective caries removal:  Make sure to choose a dentist who is experienced in this technique.  Be sure to ask your dentist any questions you have about the procedure.  Follow your dentist's instructions after the procedure, such as brushing and flossing your teeth regularly and avoiding sticky foods.  With proper care, your teeth can remain healthy and strong for many years to come. | 1. [According to FDI, the recommended clinical guidelines for the removal of carious tissue in dentine cavities and symptomless teeth are as follows1](https://www.fdiworlddental.org/deep-dentine-caries-and-restorative-care): 2. Preserve non-demineralized and re-mineralizable dentine. 3. Achieve an adequate seal by placing the peripheral restoration onto sound dentine and/or enamel, thus controlling the carious lesion and inactivating remaining bacteria. 4. Minimize discomfort/pain and dental anxiety.   [The treatment options for carious tissue removal include**^2^**](https://link.springer.com/article/10.1007/s41894-019-0042-z)[**^3^**](https://pubmed.ncbi.nlm.nih.gov/27099358/):   - Deep carious lesion in a tooth with a vital pulp - Non-selective removal to hard or firm dentin (no longer recommended) - Stepwise removal - Selective removal to soft dentin - No removal, involving non-restorative cavity management, fissure sealing, or—as shown—the Hall Technique (Hall Technique restricted to primary teeth)   I hope this helps!  **Learn more:**  [1. fdiworlddental.org](https://www.fdiworlddental.org/deep-dentine-caries-and-restorative-care)[2. link.springer.com](https://link.springer.com/article/10.1007/s41894-019-0042-z)[3. pubmed.ncbi.nlm.nih.gov](https://pubmed.ncbi.nlm.nih.gov/27099358/) | Schwendicke F, Frencken JE, Bjørndal L, Maltz M, Manton DJ, Ricketts D, Van Landuyt K, Banerjee A, Campus G, Doméjean S, Fontana M, Leal S, Lo E, Machiulskiene V, Schulte A, Splieth C, Zandona AF, Innes NP. Managing Carious Lesions: Consensus Recommendations on Carious Tissue Removal. Adv Dent Res. 2016 May;28(2):58-67. doi: 10.1177/0022034516639271. PMID: 27099358.  Banerjee A, Frencken JE, Schwendicke F, Innes NPT. Contemporary operative caries management: consensus recommendations on minimally invasive caries removal. Br Dent J. 2017 Aug 11;223(3):215-222. doi: 10.1038/sj.bdj.2017.672. PMID: 28798430.  ICCMS Guide for Practitioners and Educators  https://www.iccms-web.com/uploads/asset/59284654c0a6f822230100.pdf  Summary of evidence: Selective caries removal protocol. Remove all caries to hard dentin at DEJ. Remove all caries to firm dentin. Leave soft dentin if needed to avoid pulp exposure. |
| 5. What is the material of choice for direct pulp capping (vital pulp therapy)?  Discipline: Endodontology/Operative Dentistry | The material of choice for direct pulp capping in vital pulp therapy may vary depending on the dentist's preference, but traditionally, calcium hydroxide has been the most commonly used material. Other materials that have been used successfully include mineral trioxide aggregate (MTA), bioactive glasses, and calcium-enriched mixture (CEM) cement. The choice of material will depend on the individual case and the dentist's experience with different materials. It's important to note that the success of direct pulp capping also depends on factors such as the size and location of the pulp exposure, the amount of remaining dentin, and the patient's overall oral health. | As of my last knowledge update in September 2021, the material of choice for direct pulp capping in vital pulp therapy was calcium hydroxide or mineral trioxide aggregate (MTA). Calcium hydroxide has been a traditional choice due to its antibacterial properties and ability to stimulate dentin bridge formation. MTA, on the other hand, has gained popularity because of its superior sealing ability, biocompatibility, and ability to promote dentin bridge formation and pulp cell survival.  Please note that dental materials and best practices may have evolved since my knowledge update. It is always best to consult with a dental professional for the most current information on dental materials and procedures. | The material of choice for direct pulp capping (vital pulp therapy) is mineral trioxide aggregate (MTA). MTA is a cementitious material that has been shown to be biocompatible, antibacterial, and moisture-resistant. It is also radiopaque, which makes it easy to see on x-rays. MTA has been shown to have a high success rate in clinical trials, and it is becoming the preferred material for direct pulp capping.  Other materials that have been used for direct pulp capping include calcium hydroxide and zinc oxide-eugenol. However, these materials have not been shown to be as effective as MTA. Calcium hydroxide is a basic material that can irritate the pulp, and zinc oxide-eugenol is an irritant that can kill the pulp.  The success of direct pulp capping depends on a number of factors, including the size of the pulp exposure, the health of the pulp, and the skill of the dentist. However, MTA has been shown to be a safe and effective material for direct pulp capping, and it is the preferred material for this procedure.  Here are some additional details about MTA:  MTA is a mixture of tricalcium silicate, dicalcium silicate, and tricalcium aluminate.  MTA is radiopaque, which makes it easy to see on x-rays.  MTA is biocompatible, meaning that it is not harmful to the body.  MTA is antibacterial, meaning that it can kill bacteria.  MTA is moisture-resistant, meaning that it can withstand moisture without dissolving.  MTA sets slowly, which gives the dentist time to place it properly.  MTA is relatively inexpensive.  If you have a tooth with a small pulp exposure, your dentist may recommend direct pulp capping with MTA. This procedure is a safe and effective way to save your tooth and prevent the need for a root canal. | [Calcium hydroxide is generally used as the main material for direct pulp capping1](https://www.sciencedirect.com/topics/engineering/capping-material). [It was first introduced to dentistry in 1921 and is probably the most highly regarded of the available materials1](https://www.sciencedirect.com/topics/engineering/capping-material). [It gives excellent clinical results and is currently typically used in association with some sort of glass-ionomer cement1](https://www.sciencedirect.com/topics/engineering/capping-material). [Zinc oxide eugenol (ZOE) is another commonly used material in dentistry2](https://en.wikipedia.org/wiki/Pulp_capping).  I hope this helps! Let me know if you have any other questions. | European Society of Endodontology (ESE) developed by:; Duncan HF, Galler KM, Tomson PL, Simon S, El-Karim I, Kundzina R, Krastl G, Dammaschke T, Fransson H, Markvart M, Zehnder M, Bjørndal L. European Society of Endodontology position statement: Management of deep caries and the exposed pulp. Int Endod J. 2019 Jul;52(7):923-934. doi: 10.1111/iej.13080. PMID: 30664240.  <https://www.aae.org/wp-content/uploads/2021/05/VitalPulpTherapyPositionStatement_v2.pdf>  Summary of evidence:  ESE: Several recent studies have confirmed high levels of success after non-selective (complete) caries removal, leading to pulp exposure, and an enhanced vital pulp therapy procedure involving the use of a hydraulic calcium silicate cement (not calcium hydroxide), an operating micro- scope (Bogen et al. 2008, Marques et al. 2015) and/or the use of a disinfection agent.  In all cases, a hydraulic calcium silicate cement should be placed directly onto the pulp tissue and the tooth definitively restored immediately to prevent further micro-leakage.  Hydraulic calcium silicate materials, such as mineral trioxide aggregate (MTA), have demonstrated superior histological (Aeinehchi et al. 2003, Nair et al. 2008) and clinical outcomes compared with calcium hydroxide in treatment of the exposed pulp.  AEE: Calcium silicate-based cements are a class of materials that include tricalcium silicates, dicalcium silicates, hydraulic calcium silicate cements, and “bioceramics.” Clinical outcomes have demonstrated consistent success with these materials and mineral trioxide aggregate (MTA) is one of many tricalcium silicates that is widely used and the most extensively studied. When MTA and other Calcium silicate-based cements are used for vital pulp therapy procedures in permanent teeth with symptomatic or asymptomatic irreversible pulpitis, success rates range from 85-100% at 1-2 years. However, it is noteworthy that calcium hydroxide, glass ionomer cements (GICs) and resin- based materials trail in clinical outcomes and demonstrate a lower range of success varying from 43% - 92%. |
| 6. What radiographs and how often should be prescribed for an adult recall patient with clinical caries or at increased risk for caries? What radiographs and how often should be prescribed for an adult recall patient with no clinical caries and with no increased risk for caries?  Discipline: Oral and Maxillofacial Radiology | The frequency and type of radiographs prescribed for an adult recall patient depend on their individual dental needs and risk factors. The American Dental Association (ADA) has established guidelines to help dental professionals determine the appropriate imaging for their patients.  For an adult recall patient with clinical caries or at increased risk for caries, the following radiographs are typically prescribed:  Bitewing radiographs: These are the most commonly used radiographs for detecting dental caries in the posterior teeth. Depending on the patient's risk level, the ADA recommends taking bitewing radiographs every 6-12 months.  Periapical radiographs: These radiographs are used to examine the entire tooth structure, from the crown to the root. They are useful for detecting dental caries, as well as other dental conditions such as abscesses and fractures. Depending on the patient's risk level and dental history, periapical radiographs may be taken every 3-5 years.  For an adult recall patient with no clinical caries and no increased risk for caries, the following radiographs may be prescribed:  Bitewing radiographs: The ADA recommends taking bitewing radiographs every 12-24 months for patients with low caries risk.  Panoramic radiographs: These radiographs provide a panoramic view of the entire oral cavity, including the teeth, jaws, and surrounding structures. They are useful for detecting impacted teeth, cysts, tumors, and other conditions that may not be visible with other types of radiographs. Depending on the patient's risk level and dental history, panoramic radiographs may be taken every 3-5 years.  It is important to note that the frequency and type of radiographs may vary depending on the patient's age, dental history, and risk factors. Dental professionals should use their clinical judgment and follow the ADA guidelines to determine the appropriate imaging for each individual patient. | I am not a dentist, but I can provide some general information based on existing guidelines. It is essential to consult with a licensed dental professional for individualized recommendations.  For an adult recall patient with clinical caries or at increased risk for caries, the American Dental Association (ADA) and the Food and Drug Administration (FDA) have offered general guidelines that may apply:   1. Bitewing radiographs: These can be taken at 6-18 month intervals, depending on the specific needs of the patient and the extent of their caries risk. 2. Periapical radiographs: These may be recommended if the dentist needs to assess the root structure or look for any signs of infection or bone loss. 3. Panoramic radiographs or full mouth series (FMX): These are not typically prescribed for routine recall visits but may be considered if the patient has extensive dental needs or if there has been a significant change in their dental or medical history.   For an adult recall patient with no clinical caries and no increased risk for caries, the guidelines may differ:   1. Bitewing radiographs: These can be taken at 24-36 month intervals, as they help to identify early interproximal caries and monitor existing restorations. 2. Periapical radiographs: These are typically not needed for routine recall visits unless there is a specific concern or need. 3. Panoramic radiographs or full mouth series (FMX): These are not routinely prescribed for patients with no clinical caries or increased risk for caries. They may be considered on a case-by-case basis, depending on the patient's dental or medical history and specific concerns.   Remember that these are general guidelines and may not apply to every patient. Dentists should always use their professional judgment and consider factors such as the patient's age, medical and dental history, and individual risk factors when determining the appropriate radiographs and frequency for each patient. | Adult recall patient with clinical caries or at increased risk for caries  Posterior bitewing radiographs every 6-12 months.  Periapical radiographs may be indicated for patients with deep caries, large restorations, or suspected root fractures.  Panoramic radiographs may be indicated for patients with missing teeth, suspected impacted teeth, or temporomandibular joint (TMJ) problems.  Adult recall patient with no clinical caries and with no increased risk for caries  Posterior bitewing radiographs every 24-36 months.  Periapical radiographs and panoramic radiographs are not routinely indicated for patients in this category.  It is important to note that these are just guidelines, and the frequency of radiographs may need to be adjusted based on the individual patient's needs. For example, a patient with a history of rapid caries progression may need more frequent radiographs, while a patient with a very low risk of caries may be able to go longer between radiographs.  It is also important to discuss the risks and benefits of radiographs with your dentist before any radiographs are taken. | [According to the American Dental Association (ADA), a full-mouth intraoral radiographic examination is preferred when the patient has clinical evidence of generalized oral disease or a history of extensive dental treatment1](https://jada.ada.org/article/S0002-8177(21)00172-0/fulltext). [For recall patients with clinical caries or at increased risk of developing caries, posterior bitewing exams are recommended at 6- to 18-month intervals2](https://www.rdhmag.com/patient-care/radiology/article/16407091/ada-guidelines-coding-benefits-for-radiographs). [For recall patients with no clinical caries and not at increased risk for caries, posterior bitewing exams are recommended at 24- to 36-month intervals2](https://www.rdhmag.com/patient-care/radiology/article/16407091/ada-guidelines-coding-benefits-for-radiographs).  I hope this helps!  **Learn more:**  [1. jada.ada.org](https://jada.ada.org/article/S0002-8177(21)00172-0/fulltext)[2. rdhmag.com](https://www.rdhmag.com/patient-care/radiology/article/16407091/ada-guidelines-coding-benefits-for-radiographs)[3. ada.org](https://www.ada.org/-/media/project/ada-organization/ada/ada-org/files/resources/research/hpi/alara_poster_2018.pdf?rev=57eaaa0b1de74f1091effe134a289a47&hash=EF4A719AA46569B2DCE83ED243299C21) | <https://www.fda.gov/media/84818/download>  Summary of evidence: For increased risk of caries: 6-18 months.  For low risk of caries 24-36 months. |
| 7. What are the indications to remove wisdom teeth?  Discipline: Oral Surgery | Wisdom teeth are the last molars to emerge at the back of the mouth, typically appearing in the late teens or early twenties. While some people have enough room in their mouths to accommodate their wisdom teeth, others may experience issues that require their removal.  The indications for wisdom teeth removal may include:  Impacted teeth: When a wisdom tooth does not have enough room to emerge properly, it may become impacted, which means that it is trapped beneath the gum line. This can cause pain, swelling, and infection.  Crowding: If there is not enough space in the mouth for the wisdom teeth to emerge, they can push other teeth out of alignment, causing crowding and discomfort.  Infection or inflammation: Wisdom teeth that have partially emerged can create a flap of gum tissue that can trap food and bacteria, leading to infection or inflammation.  Tooth decay: Wisdom teeth that are difficult to clean may be more prone to decay.  Cysts or tumors: In rare cases, cysts or tumors can form around impacted wisdom teeth, potentially causing damage to the jawbone, nerves, or other teeth.  If you are experiencing any of these issues with your wisdom teeth, you should consult with a dentist or oral surgeon to determine if removal is necessary. | Wisdom teeth, also known as third molars, are the last teeth to develop and emerge in the mouth. They usually come in between the ages of 17 and 25. Not everyone needs to have their wisdom teeth removed, but there are some indications for removal. These include:   1. Impacted wisdom teeth: If the teeth do not have enough room to fully emerge, they can become impacted, causing pain, swelling, and infection. 2. Infection or gum disease: Impacted or partially erupted wisdom teeth can create a flap of gum tissue that traps food particles and bacteria, leading to infection or gum disease. 3. Tooth decay: Wisdom teeth can be difficult to clean due to their location, making them more susceptible to tooth decay. 4. Damage to neighboring teeth: Impacted wisdom teeth can push against adjacent teeth, causing damage or misalignment. 5. Cysts or tumors: In rare cases, impacted wisdom teeth can lead to the development of cysts or tumors in the jawbone. 6. Orthodontic concerns: In some cases, wisdom teeth can interfere with orthodontic treatment or cause crowding of other teeth. 7. Pain or discomfort: Wisdom teeth can cause pain or discomfort in the mouth, jaw, or surrounding areas.   Ultimately, the decision to remove wisdom teeth should be made in consultation with a dental professional, who will evaluate the individual's specific circumstances and determine the best course of action. | Wisdom teeth are the four teeth that are located at the back of your mouth, one on each side of the top and bottom jaw. They are the last teeth to come in, usually between the ages of 17 and 25. Wisdom teeth are not always necessary, and in some cases, they can cause problems.  There are a number of indications for removing wisdom teeth. These include:  Impaction: This is when the wisdom tooth is unable to grow in properly and becomes stuck under the gum tissue or bone. Impacted wisdom teeth can cause pain, infection, and other problems.  Crowding: If there is not enough room in your mouth for all of your teeth, your wisdom teeth may push your other teeth out of alignment. This can lead to problems with chewing, speaking, and tooth decay.  Infection: Wisdom teeth can become infected, which can lead to pain, swelling, and fever. In some cases, infection can spread to the jawbone or other parts of the body.  Cysts: Wisdom teeth can sometimes form cysts around them. These cysts can damage the surrounding bone and teeth.  Gum disease: Wisdom teeth that are difficult to clean can be more likely to develop gum disease. Gum disease can cause pain, bleeding, and tooth loss.  If you have any of these problems, you may need to have your wisdom teeth removed. The decision of whether or not to remove your wisdom teeth is a personal one, and you should discuss it with your dentist. | [Wisdom teeth are usually removed if they cause problems such as pain, infection, or damage to other teeth1](https://www.mayoclinic.org/tests-procedures/wisdom-tooth-extraction/about/pac-20395268). [Some dentists recommend removing wisdom teeth if they don’t fully emerge2](https://www.mayoclinic.org/diseases-conditions/wisdom-teeth/expert-answers/wisdom-teeth-removal/faq-20058558). [Many dentists believe it’s better to remove wisdom teeth at a younger age, before the roots and bone are fully formed, and when recovery is generally faster after surgery2](https://www.mayoclinic.org/diseases-conditions/wisdom-teeth/expert-answers/wisdom-teeth-removal/faq-20058558). [People of all ages can have their wisdom teeth removed3](https://my.clevelandclinic.org/health/treatments/22119-wisdom-teeth-removal). A general guide is to remove wisdom teeth between 18-21, but it is really up to the assessment of the oral surgeon to decide when is the best timing after a thorough diagnosis including imaging such as X-rays[4](https://www.verywellhealth.com/how-are-wisdom-teeth-removed-1059378).  I hope that helps! Let me know if you have any other questions.  **Learn more:**  [1. mayoclinic.org](https://www.mayoclinic.org/tests-procedures/wisdom-tooth-extraction/about/pac-20395268)[2. mayoclinic.org](https://www.mayoclinic.org/diseases-conditions/wisdom-teeth/expert-answers/wisdom-teeth-removal/faq-20058558)[3. my.clevelandclinic.org](https://my.clevelandclinic.org/health/treatments/22119-wisdom-teeth-removal)[4. verywellhealth.com](https://www.verywellhealth.com/how-are-wisdom-teeth-removed-1059378)[5. bing.com](https://bing.com/search?q=When+to+remove+wisdom+teeth%3f)+2 more | Parameters of Care: AAOMS Clinical Practice Guidelines for Oral and Maxillofacial Surgery, Sixth Edition, J Oral Maxillofac Surg 75:e50-e73, 2017, Suppl 1  https://www.nice.org.uk/guidance/ta1/resources/guidance-on-the-extraction-of-wisdom-teeth-pdf-63732983749  <https://www.mouthhealthy.org/all-topics-a-z/wisdom-teeth>  https://www.aaoms.org/docs/govt_affairs/advocacy_white_papers/management_third_molar_white_paper.pdf  <https://www.aaoms.org/images/uploads/pdfs/management_third_molar_supporting_information.pdf>  Summary of evidence:  1) Dental caries  2) Pericoronitis 3) Root resorption  4) Periodontitis  5) Infections (local and fascial space)  6) Cysts 7) Tumors 8) Mandible fractures. |
| 8. What is the recommended age for a child's first dental visit?  Discipline: Pediatric Dentistry | The American Academy of Pediatric Dentistry (AAPD) recommends that children have their first dental visit by their first birthday or within six months of the eruption of their first tooth, whichever comes first. This early visit is important for the dentist to check the child's oral health and development, and for parents to learn about proper oral care for their child. Subsequent visits should occur every six months, or as recommended by the child's dentist. | The American Dental Association (ADA) and the American Academy of Pediatric Dentistry (AAPD) recommend that a child's first dental visit should occur within six months after the eruption of their first tooth, or by their first birthday, whichever comes first. Establishing a dental home early can help promote good oral hygiene habits, monitor dental development, and identify and address any issues early on. It's important to maintain regular dental checkups every six months or as recommended by your dentist. | The American Academy of Pediatric Dentistry (AAPD) recommends that children see a dentist by the first birthday, or within 6 months of the first tooth coming in. This is because tooth decay can start even before a child's first tooth appears, and early detection and treatment can help prevent cavities.  The first dental visit is usually a short appointment where the dentist will:  Meet the child and family. The dentist will get to know the child and their family, and answer any questions they may have about dental care.  Examine the child's teeth and gums. The dentist will look for any signs of tooth decay, gum disease, or other problems.  Provide information on oral health care. The dentist will teach the child and parents how to brush and floss their teeth properly, and how to choose healthy foods.  The first dental visit can be a new and exciting experience for both the child and the parents. By starting early, you can help your child develop a lifelong love of good oral health. | [According to the American Academy of Pediatric Dentistry (AAPD) and the American Academy of Pediatrics, children should have their first dental visit by age 1 or within six months after their first tooth erupts, whichever comes first1](https://www1.deltadentalins.com/wellness/kids/articles/first-visit-to-dentist.html). [This visit will establish a dental home for your child and help prevent or treat any developing problems1](https://www1.deltadentalins.com/wellness/kids/articles/first-visit-to-dentist.html).  Is there anything else you would like to know?  Learn more:  [1. www1.deltadentalins.com](https://www1.deltadentalins.com/wellness/kids/articles/first-visit-to-dentist.html)[2. stanfordchildrens.org](https://www.stanfordchildrens.org/en/topic/default?id=a-childs-first-dental-visit-fact-sheet-1-1509)[3. my.clevelandclinic.org](https://my.clevelandclinic.org/health/articles/10965-your-childs-first-dental-visit)[4. utknoxvilledentists.com](https://utknoxvilledentists.com/whats-the-right-age-for-a-childs-first-dentist-visit/)[5. urmc.rochester.edu](https://www.urmc.rochester.edu/encyclopedia/content.aspx?contenttypeid=1&contentid=1509)+3 more | <https://www.aapd.org/assets/1/7/DentalHomeNeverTooEarly.pdf>  <https://www.nhs.uk/live-well/healthy-teeth-and-gums/taking-care-of-childrens-teeth/>  Summary of evidence: When the first primary tooth appears and up to 12 months of age. |
| 9. What is recommended to arrest a cavitated caries lesion in primary teeth?  Discipline: Pediatric Dentistry | Arresting cavitated caries lesions in primary teeth requires a multi-faceted approach that includes the removal of caries, the use of fluoride, and the promotion of good oral hygiene habits. The following are some recommended strategies:   1. Remove caries: The first step in arresting cavitated caries lesions is to remove the decayed tooth structure. This can be done using a drill or laser. The goal is to remove all the decayed tissue and leave only healthy tooth structure. In some cases, a dental filling may be necessary to restore the tooth's structure. 2. Fluoride application: Fluoride is a mineral that can help to strengthen teeth and prevent further decay. It is recommended to apply fluoride varnish or a fluoride-containing product to the affected tooth after removing the decayed tissue. This can help to remineralize the tooth and prevent the progression of the lesion. 3. Promote good oral hygiene: Encouraging good oral hygiene habits, such as brushing twice a day and flossing daily, can help to prevent the formation of new cavities. Additionally, reducing sugary foods and drinks in the diet can also help to prevent further decay. 4. Regular dental check-ups: It is important for children to have regular dental check-ups every six months to monitor the progression of any cavitated caries lesions and to ensure that the teeth and gums are healthy.   It is important to note that the effectiveness of these strategies can depend on the severity of the lesion and the child's individual circumstances. A dental professional can provide personalized recommendations for treating cavitated caries lesions in primary teeth. | I'm not a dentist, but I can provide some general information on managing cavitated caries lesions in primary teeth. It is important to consult with a dental professional for personalized advice and treatment.  To arrest a cavitated caries lesion in primary teeth, dental professionals may consider the following options:   1. Atraumatic Restorative Treatment (ART): This involves removing the decayed tooth structure with hand instruments and filling the cavity with a restorative material, typically glass ionomer cement (GIC), which has fluoride-releasing properties to help prevent further decay. 2. Silver Diamine Fluoride (SDF): SDF is a topical antimicrobial and remineralizing agent that can help arrest caries by inhibiting bacterial growth and promoting remineralization of the tooth structure. It may cause black discoloration of the treated area, but it's an effective and minimally invasive option, especially for children who have difficulty cooperating in a dental setting. 3. Dental sealants: In some cases, dental professionals may use sealants to cover and protect the tooth surface from further decay. Sealants are typically applied to the pits and fissures of molars to prevent bacteria and food debris from accumulating. 4. Fluoride varnish: Fluoride varnish can be applied to the tooth surface to strengthen the enamel and help prevent further decay. This is a common preventive measure in pediatric dentistry. 5. Proper oral hygiene and dietary habits: Encouraging children to maintain good oral hygiene practices, like brushing twice a day with a fluoride toothpaste and flossing daily, along with a balanced diet low in sugar, can help prevent the progression of cavities. 6. Regular dental check-ups: Regular dental check-ups allow the dentist to monitor the lesion and provide timely intervention if needed.   Ultimately, the choice of treatment depends on the size and location of the cavity, the child's age, and their cooperation level. Consult with a dental professional to determine the most appropriate treatment for a specific situation. | The best way to arrest a cavitated caries lesion in primary teeth is to use silver diamine fluoride (SDF). SDF is a liquid solution that contains silver nitrate and fluoride. It is applied to the tooth surface with a small brush or cotton swab. SDF works by killing the bacteria that cause tooth decay and by providing fluoride to the tooth, which helps to strengthen the enamel and prevent further decay.  SDF is a safe and effective treatment for arresting cavitated caries lesions in primary teeth. It is less expensive than traditional dental fillings, and it does not require any drilling or anesthesia. SDF is also a good option for children who are at high risk for tooth decay, such as those who have special needs or who live in areas with poor oral health care.  If you have a cavitated caries lesion in your child's primary tooth, talk to your dentist about whether SDF is a good option for your child.  Here are some additional tips for preventing and arresting tooth decay in primary teeth:   - Brush your child's teeth twice a day with fluoride toothpaste. - Floss your child's teeth once a day. - Make sure your child gets regular dental checkups and cleanings. - Limit your child's intake of sugary drinks and snacks. - Encourage your child to drink water instead of sugary drinks. - Teach your child how to brush and floss their teeth properly. - Set a good example by brushing and flossing your own teeth regularly. | [According to an expert panel convened by the American Dental Association Council on Scientific Affairs and the Center for Evidence-Based Dentistry, to arrest advanced cavitated carious lesions on any coronal surface of primary teeth, clinicians should prioritize the use of 38% silver diamine fluoride (SDF) solution (biannual application) over 5% sodium fluoride varnish (application once per week for 3 weeks)1](https://jada.ada.org/article/S0002-8177%2818%2930469-0/fulltext). [These approaches have shown to be effective in arresting or reversing carious lesions1](https://jada.ada.org/article/S0002-8177%2818%2930469-0/fulltext).  Learn more:  [1. jada.ada.org](https://jada.ada.org/article/S0002-8177%2818%2930469-0/fulltext)[2. ncbi.nlm.nih.gov](https://www.ncbi.nlm.nih.gov/books/NBK573067/)[3. jada.ada.org](https://jada.ada.org/article/S0002-8177%2818%2930469-0/pdf)[4. oralhealth.cochrane.org](https://oralhealth.cochrane.org/news/interventions-treating-cavitated-or-dentine-carious-lesions) | American Academy of Pediatric Dentistry. Fluoride therapy. The Reference Manual of Pediatric Dentistry. Chicago, Ill.: American Academy of Pediatric Dentistry; 2022:317-20.  Summary of evidence: Silver Diamine Fluoride (SDF). |
| 10. What tooth is the most bilaterally symmetrical in the human permanent dentition?  Discipline: Dental Morphology/Operative Dentistry/Prosthodontics | The human permanent central incisor is the most bilaterally symmetrical tooth in the human dentition. This is because it has a single root and a simple crown with a straight, symmetrical incisal edge that divides the crown into two equal halves. The central incisor is located in the front of the mouth, and there is one on each side of the maxillary and mandibular dental arches.  Therefore, both the maxillary and mandibular central incisors are equally bilaterally symmetrical. | In the human permanent dentition, the most bilaterally symmetrical teeth are the central incisors. There are two central incisors in the upper jaw (maxillary central incisors) and two in the lower jaw (mandibular central incisors). These teeth have the same shape and size on both sides of the dental arch, making them the most symmetrical teeth in the human mouth. | The most bilaterally symmetrical tooth in the human permanent dentition is the maxillary central incisor. This tooth has one root and one canal, and the configuration of these structures is the same on both sides of the mouth in 100% of cases.  The maxillary lateral incisor is also very symmetrical, with 99.2% of cases showing bilateral symmetry. The mandibular central incisor is also very symmetrical, with 98.9% of cases showing bilateral symmetry.  The other teeth in the human permanent dentition are less symmetrical. The mandibular lateral incisor is symmetrical in 97.7% of cases, the maxillary first premolar is symmetrical in 91.2% of cases, the maxillary second premolar is symmetrical in 85.3% of cases, the mandibular first molar is symmetrical in 83.3% of cases, the mandibular second molar is symmetrical in 76.7% of cases, and the maxillary third molar is symmetrical in only 66.7% of cases.  The reasons for the variation in bilateral symmetry among teeth are not fully understood. However, it is thought that genetic factors may play a role, as well as the environment in which the teeth develop. | [According to a source I found**^1^**](https://www.calendar-uk.co.uk/faq/which-tooth-is-most-symmetrical), the lower central incisor is the smallest and most symmetrical tooth of the permanent dentition. Its anatomical elements, such as grooves and ridges, are the least evident. Is there anything else you would like to know? | Scheid RC, Weiss G. Woelfel’s Dental Anatomy, 9^th^ edition; Wolters Kluwer, 2017, Philadelphia, PA, USA. ISBN: 9781496320223  Nelson S. Wheeler’s Dental Anatomy, Physiology and Occlusion, 10^th^ edition; Elsevier, 2015, St. Louis, MI, USA. ISBN: 978-0-323-26323-8  Summary of evidence: Mandibular permanent central incisor. |
| 11. What is the ideal apical terminus of shaping and obturation of the root canal during endodontic treatment?  Discipline: Endodontology | The ideal apical terminus of shaping and obturation of the root canal during endodontic treatment is a matter of ongoing debate and varies among different endodontic schools of thought. However, most current guidelines suggest that the apical constriction, also known as the apical foramen, should be used as the apical terminus of shaping and obturation.  The apical constriction is the narrowest part of the root canal, located at the apical end of the root, and represents the physiological limit of the pulp tissue. The aim of root canal shaping is to prepare the canal to the apical constriction, while preserving the integrity of the apical tissues and avoiding over-instrumentation and perforation.  The ideal apical terminus of obturation is also the apical constriction, or within 0.5 to 1 mm short of it, depending on the filling technique used. The obturation material should be tightly condensed to create a fluid-tight seal and prevent bacterial microleakage.  It's important to note that the choice of the apical terminus depends on various factors such as the patient's anatomy, the type of instrumentation and obturation techniques used, and the clinician's experience and preferences. Therefore, the ideal apical terminus may vary depending on the situation. | The ideal apical terminus of shaping and obturation during endodontic treatment is the cemento-dentinal junction (CDJ) or the minor constriction of the root canal, which is also known as the apical constriction. This area is located within 0.5 to 1 mm short of the radiographic apex, which is the anatomical tip of the root. Achieving this ideal apical terminus helps in sealing the canal effectively, preventing bacterial leakage and promoting periapical healing.  However, it is important to note that the exact location of the apical constriction can vary between individuals and even between different teeth in the same individual. Therefore, it is crucial for the clinician to use various diagnostic tools, such as electronic apex locators, radiographs, and tactile sensation to accurately determine the working length, which corresponds to the ideal apical terminus.  Proper shaping and obturation of the root canal system are essential for successful endodontic treatment. Proper shaping allows for efficient disinfection of the canal, while obturation with a biocompatible material, such as gutta-percha and sealer, helps to prevent reinfection and promote healing of the periapical tissues. | The ideal apical terminus of shaping and obturation of the root canal during endodontic treatment is at or within 2 mm of the radiographic apex. This is because bacteria and their byproducts, as well as infected dentinal debris may remain in the most apical portion of the canal; these irritants may jeopardize apical healing.  There are a few different methods for determining the working length, or the distance from the coronal reference point to the point at which canal preparation and obturation should terminate. The most clinically relevant landmark is the apical constriction, whether in dentin or cementum. This has the narrowest diameter of the canal thus the most rational point at which to terminate canal preparation.  Other methods for determining the working length include:   - Electronic apex locators - Radiographic techniques - Tactile sensation   Once the working length has been determined, the canal should be prepared to a tapered funnel shape. This allows adequate cleaning and distortion of obturation materials by compacting. The apical extent of the canal system should be clean, patent, and completely obturated. The shape and position of the apical foramen should remain unchanged.  Obturation is the process of filling the root canal system with a material that will prevent the reinfection of the pulp space by micro-organisms from the oral cavity. There are a variety of obturation materials available, including gutta-percha, silver points, and resin-based sealers.  The success of endodontic treatment depends on a number of factors, including the skill of the dentist, the type of infection, and the anatomy of the tooth. However, by following the principles of cleaning, shaping, and obturation, dentists can increase the chances of a successful outcome.  Sources  [info](https://bard.google.com/faq#citation)  1.  [fdocuments.in/document/rct-procedure.html](https://fdocuments.in/document/rct-procedure.html)  2.  [www.researchgate.net/publication/12683300_Apical_terminus_of_root_canal_treatment_procedures](https://www.researchgate.net/publication/12683300_Apical_terminus_of_root_canal_treatment_procedures)  3.  [www.libertyendo.com/files/2014/06/Instrumentation.pdf](https://www.libertyendo.com/files/2014/06/Instrumentation.pdf) | The apical constriction (AC) is commonly advocated as the ideal termination for root canal treatment (RCT), being a natural narrowing of the root canal and almost at the termination of the pulp. This is supposedly where an apical stop is formed, against which the obturation materials are packed. [Because this constriction is usually not present, the apical foramen (AF) may be a more useful landmark1](https://www.sciencedirect.com/science/article/pii/S1079210400800232).  [The original position of the apical foramen should be maintained during root canal preparation2](https://www.nature.com/articles/sj.bdj.2014.203). The foraminal size of the root canal should be gauged and enlarged to allow for adequate cleaning and shaping[3](https://www.nature.com/articles/sj.bdj.2014.206). | European Society of Endodontology. Quality guidelines for endodontic treatment: consensus report of the European Society of Endodontology. Int Endod J. 2006 Dec;39(12):921-30. doi: 10.1111/j.1365-2591.2006.01180.x. PMID: 17180780.  Summary of evidence: The objective of determining the working length is to enable the root canal to be prepared as close to the apical constriction as possible. The location of the apical constriction normally varies between 0.5 and 2 mm from the radiographic apex. Recommended methods are electronic and radiographic. |
| 12. In surgical extrusion and intentional replantation, what is the maximum duration of extra alveolar manipulation to prevent damage of the periodontal ligament?  Discipline: Endodontology | The extra-alveolar time is a critical factor in the success of surgical extrusion and intentional replantation procedures. The goal is to minimize the extra-alveolar time as much as possible to prevent damage to the periodontal ligament (PDL).  The maximum duration of extra-alveolar manipulation depends on various factors such as the type of tooth, the extent of trauma, and the patient's age and overall health. In general, the extra-alveolar time should be limited to 15-20 minutes to prevent irreversible damage to the PDL.  However, it is important to note that the success of these procedures also depends on other factors such as proper case selection, adequate anesthesia and analgesia, appropriate instrumentation, and careful post-operative management. Therefore, it is crucial to consult with a qualified dental professional to determine the best course of action for your specific case. | Surgical extrusion and intentional replantation are dental procedures where a tooth is deliberately removed from its socket and then reinserted. The periodontal ligament (PDL) is a key factor in the success of these procedures, as it contains cells and fibers necessary for tooth reattachment and healing.  The maximum duration of extra-alveolar manipulation to minimize damage to the PDL and maximize the chances of successful replantation varies based on different factors and the specific context of each case. However, it is generally recommended to minimize the extra-alveolar time as much as possible.  A commonly cited guideline is to keep the tooth out of the socket for no more than 15-20 minutes to minimize damage to the PDL. The success rate tends to decrease significantly if the extra-alveolar time exceeds 60 minutes. During the extra-alveolar period, it is crucial to keep the PDL moist, for instance by placing the tooth in a physiological storage medium, such as Hank's balanced salt solution (HBSS), or milk, to prevent the PDL cells from drying out and dying.  It's essential to consult a dental professional to discuss the specifics of each case and the best approach to minimize PDL damage during surgical extrusion or intentional replantation. | The maximum duration of extra alveolar manipulation to prevent damage of the PDL in surgical extrusion and intentional replantation is 10 minutes. Any manipulation beyond this time period is likely to cause further damage to the PDL and decrease the chances of successful replantation.  The PDL is a fibrous connective tissue that surrounds the root of a tooth and anchors it to the alveolar bone. It is composed of two main components: the cellular component and the extracellular matrix. The cellular component consists of fibroblasts, osteoblasts, and osteoclasts. The extracellular matrix consists of collagen fibers, ground substance, and other proteins.  The PDL plays an important role in tooth eruption, anchorage, and tooth movement. It also helps to protect the tooth from trauma and infection. During surgical extrusion and intentional replantation, the PDL can be damaged by excessive manipulation. This can lead to inflammation, necrosis, and ankylosis.  To prevent damage to the PDL, it is important to limit the duration of extra alveolar manipulation to 10 minutes. If the tooth is not replanted within this time period, the chances of success decrease significantly.  Here are some tips for preventing damage to the PDL during surgical extrusion and intentional replantation:   - Use gentle force when manipulating the tooth. - Avoid excessive movement of the tooth. - Replant the tooth as soon as possible. - Monitor the tooth for signs of infection or inflammation. | [According to a clinical review of cases undertaken at a dental hospital in India, an extra-alveolar time of more than **15 minutes** in dry conditions compromises the viability of the PDL cells, increasing the risk of replacement resorption and ankylosis1](https://www.nature.com/articles/s41415-020-1988-6).  [In general, transplanted and replanted or extruded teeth should not be moved orthodontically for at least **six months** after the procedure2](https://onlinelibrary.wiley.com/doi/full/10.1111/iej.13456).  I hope this helps! Let me know if you have any other questions. | Plotino G, Abella Sans F, Duggal MS, Grande NM, Krastl G, Nagendrababu V, Gambarini G. European Society of Endodontology position statement: Surgical extrusion, intentional replantation and tooth autotransplantation: European Society of Endodontology developed by. Int Endod J. 2021 May;54(5):655-659. doi: 10.1111/iej.13456. Epub 2021 Jan 26. PMID: 33501680.  Summary of evidence: The total extra-alveolar time of manipulation should be as short as possible and never exceed 15 min, as it is a critical factor for determining the long-term prognosis of the treatment. |
| 13. What are the indications and contraindications for administration of systemic antibiotics in endodontics?  Discipline: Endodontology | Systemic antibiotics may be indicated in endodontics for the management of bacterial infections that have spread beyond the confines of the root canal system. The following are indications for the use of systemic antibiotics in endodontics:   1. Acute apical abscess or cellulitis: When the patient presents with a severe localized or diffuse swelling accompanied by systemic symptoms like fever, malaise, and lymphadenopathy. 2. Acute exacerbation of chronic periapical periodontitis: When a chronic lesion shows acute exacerbation with increased pain, swelling, and tenderness. 3. Systemic infection: When the bacterial infection has spread to other parts of the body, such as sepsis, infective endocarditis, or brain abscess. 4. Immunocompromised patients: When the patient's immune system is compromised due to a medical condition or treatment like chemotherapy, and they are at an increased risk of systemic infection.   Contraindications for the use of systemic antibiotics in endodontics include:   1. Localized infection: If the bacterial infection is confined to the root canal system, antibiotics are not indicated, and the root canal treatment is the treatment of choice. 2. Allergy or sensitivity to the antibiotic: When the patient has a known allergy or sensitivity to the prescribed antibiotic. 3. Overuse or misuse of antibiotics: When the patient has a history of overuse or misuse of antibiotics leading to bacterial resistance. 4. Pregnancy or lactation: When the patient is pregnant or breastfeeding, the use of antibiotics should be minimized or avoided, especially in the first trimester of pregnancy.   It is important to note that the use of antibiotics should always be based on the proper diagnosis and clinical judgment of the dentist. The dentist should also consider the potential risks and benefits of antibiotics and the patient's medical history before prescribing antibiotics. | In endodontics, systemic antibiotics are used to manage and prevent bacterial infections associated with the dental pulp and periapical tissues. However, they should be used judiciously and only when indicated to minimize the risk of antibiotic resistance and adverse effects. The decision to prescribe antibiotics depends on the clinical presentation, patient medical history, and the dentist's judgment.  Indications for the use of systemic antibiotics in endodontics include:   1. Acute apical abscess: Antibiotics may be considered in cases of severe infection with systemic involvement, such as fever, malaise, lymphadenopathy, or cellulitis. 2. Rapidly progressing infections: If the infection is spreading rapidly and there are signs of systemic involvement, antibiotics can help limit the spread and prevent complications. 3. Compromised immune system: Patients with a compromised immune system (e.g., uncontrolled diabetes, HIV, chemotherapy, or organ transplant recipients) may have a reduced ability to fight off infections and may benefit from antibiotic therapy. 4. Persistent infections: In some cases, infections persist despite adequate root canal treatment and proper oral hygiene. In these situations, antibiotics may be prescribed to help manage the infection. 5. Prophylaxis: In certain situations, antibiotics may be prescribed as a preventive measure for high-risk patients, such as those with a history of infective endocarditis, prosthetic joint replacement, or other conditions that place them at a higher risk for systemic infection.   Contraindications for the use of systemic antibiotics in endodontics include:   1. Routine root canal treatment: Antibiotics are generally not indicated for routine root canal treatment in the absence of systemic symptoms or complications. 2. Pulpitis: Pulpitis, or inflammation of the dental pulp, is generally managed by local treatment methods (e.g., pulpotomy, pulpectomy) rather than systemic antibiotics. 3. Necrotic pulp with no symptoms: Antibiotics are not indicated for asymptomatic necrotic pulp, as local treatment can effectively manage the condition. 4. Allergy or hypersensitivity: Patients with a known allergy or hypersensitivity to the prescribed antibiotic or related medications should not receive the antibiotic. 5. Drug interactions: Antibiotics should not be prescribed if they are known to interact negatively with other medications the patient is taking.   It's important to remember that systemic antibiotics should be used as an adjunct to proper endodontic treatment and not as a standalone therapy. The primary treatment for endodontic infections is the elimination of the source of infection through root canal therapy or extraction, followed by proper restoration of the tooth. | Indications for systemic antibiotics in endodontics   - Signs of systemic involvement: Fever, malaise, cellulitis, lymphadenopathies - Acute apical periodontitis - Immunocompromised patients - Patients with predisposing conditions such as previous endocarditis - Traumatic dental injuries   Contraindications for systemic antibiotics in endodontics   - Allergy to penicillin or other antibiotics - Uncomplicated cases of acute apical periodontitis - Cases that respond to non-surgical endodontic treatment   It is important to note that antibiotics should only be used as adjuvant therapies in cases with evidence of systemic involvement. In the absence of systemic involvement, antibiotics have no evidence of therapeutic benefit. | [Antibiotics are frequently used by dentists and endodontists as part of routine practice in endodontic therapy1](https://www.aae.org/specialty/aae-issues-new-guidelines-use-systemic-antibiotics-endodontics/). [However, the use of systemic antibiotics generally is not indicated during endodontic treatment to alleviate pain or decrease the presence of infection2](https://jada.ada.org/article/S0002-8177(15)01097-1/fulltext). [Generally, an accurate diagnosis coupled with effective endodontic treatment will decrease the number of microbes enough to result in a healing outcome2](https://jada.ada.org/article/S0002-8177(15)01097-1/fulltext).  Antibiotics should be considered in patients having systemic diseases with compromised immunity or in patients with a localized congenital or acquired altered defence capacity, such as patients with infective endocarditis, prosthetic cardiac valves or recent prosthetic joint replacement[3](https://pubmed.ncbi.nlm.nih.gov/28005295/). Contra-indications for systemic antibiotics in Endodontics include most endodontic infections that are confined within the tooth and can be successfully managed by established local operative treatment[4](https://onlinelibrary.wiley.com/doi/full/10.1111/iej.12781).  I hope this helps! | Segura-Egea JJ, Gould K, Şen BH, Jonasson P, Cotti E, Mazzoni A, Sunay H, Tjäderhane L, Dummer PMH. European Society of Endodontology position statement: the use of antibiotics in endodontics. Int Endod J. 2018 Jan;51(1):20-25. doi: 10.1111/iej.12781. Epub 2017 Jun 14. PMID: 28436043.  Summary of evidence:   1. Acute apical abscess in medically compromised patients; 2. Acute apical abscess with systemic involvement (localized fluctuant swellings, elevated body temperature >38°C, malaise, lymphadenopathy, trismus; 3. Progressive infections (rapid onset of severe infection in <24 h, cellulitis or a spreading infection, osteomyelitis) where onward referral to oral surgeons may be necessary; 4. Replantation of avulsed permanent teeth (Hinckfuss & Messer 2009, Segura-Egea et al. 2017). In these cases, topical administration of antibiotics may also be indicated (Andersson et al. 2012). 5. Soft tissue trauma requiring treatment (e.g. sutures, debridement) (Diangelis et al. 2012). |
| 14. Which findings indicate an unfavorable outcome after endodontic treatment?  Discipline: Endodontology | There are several factors that may indicate an unfavorable outcome after endodontic treatment, including:   1. Presence of pre-operative periapical radiolucency (bone loss around the root tip) 2. Poor quality of root canal filling, such as voids, underfilling or overfilling 3. Incomplete removal of infected tissue or debris from the root canal system 4. Presence of persistent pain or swelling after treatment 5. Persistent or recurrent infection around the tooth or in the surrounding bone 6. Development of a new periapical radiolucency or widening of an existing radiolucency on follow-up radiographs.   It's important to note that even when endodontic treatment is performed to a high standard, there is still a chance of failure, and some teeth may require retreatment or extraction. Regular follow-up with a dentist or endodontist is recommended to monitor the outcome of treatment. | While I'm not a dentist, I can provide some general information on factors that may indicate an unfavorable outcome after endodontic treatment. Keep in mind that only a dental professional can give specific advice tailored to a patient's unique circumstances. Some factors that may suggest an unfavorable outcome include:   1. Persistent pain: If pain continues after the endodontic treatment, it could indicate a problem such as an unresolved infection or inadequate cleaning and sealing of the root canal system. 2. Recurring infection: If an infection reoccurs, it may be due to a missed canal, inadequate cleaning, or a complex root canal anatomy that was not properly addressed during treatment. 3. Failure to heal: If the periapical lesion does not show signs of healing or continues to grow after treatment, it may indicate persistent infection or other complications. 4. Tooth fracture: A tooth can become more brittle after root canal treatment, increasing the risk of fracture. A fractured tooth may not be restorable and could require extraction. 5. Poor quality of root canal filling: If the root canal filling material is insufficient or overextended, it can compromise the treatment outcome. 6. Inadequate coronal seal: A poor seal on the tooth's crown can allow bacteria to re-enter the root canal system, leading to reinfection. 7. Untreated accessory canals: If accessory or lateral canals are left untreated, they can harbor bacteria and cause reinfection. 8. Tooth restoration failure: If the final restoration (such as a crown) fails or is lost, the tooth may become susceptible to bacterial infection again. 9. Root resorption: Resorption of the tooth's root structure can weaken the tooth and lead to complications. 10. Patient factors: Certain medical conditions or habits, such as smoking or poorly controlled diabetes, can negatively affect the outcome of endodontic treatment.   It's essential to consult with a dental professional if you experience any issues after endodontic treatment. They can evaluate your situation and provide appropriate recommendations or treatment options. | There are a number of findings that can indicate an unfavorable outcome after endodontic treatment. These include:   - Incomplete removal of infected tissue. If all of the infected tissue is not removed during root canal treatment, the infection can persist and lead to problems such as pain, swelling, and abscesses. - Improper filling of the root canals. If the root canals are not properly filled, bacteria can still enter the tooth and cause an infection. - Fractured teeth. If a tooth is fractured during root canal treatment, the infection can spread to the surrounding bone and tissue. - Recurrent infection. Even if the root canal treatment is successful, there is a small risk of the infection coming back. This is more likely to happen if the tooth is damaged or if the patient has a compromised immune system.   If you experience any of the following symptoms after root canal treatment, it is important to see your dentist right away:   - Pain - Swelling - Fever - Pus draining from the tooth - Sensitivity to hot or cold - Loose teeth   If you are concerned about the outcome of your root canal treatment, talk to your dentist. They can discuss your individual risk factors and recommend ways to reduce the risk of complications. | [According to a study**^1^**](https://www.sciencedirect.com/science/article/pii/S0099239920309304), factors that can lead to unfavorable outcomes after endodontic treatment include:  Existing preoperative periapical lesion  Lack of proper permanent restoration on the treated tooth  Post placed after root canal treatment  [Complaints of new, persistent, or worsening symptoms after root canal treatment has been completed may also indicate failure2](https://pocketdentistry.com/endodontic-treatment-outcomes/).  I hope this helps! Let me know if you have any other questions. | European Society of Endodontology. Quality guidelines for endodontic treatment: consensus report of the European Society of Endodontology. Int Endod J. 2006 Dec;39(12):921-30. doi: 10.1111/j.1365-2591.2006.01180.x. PMID: 17180780.  Summary of evidence:  Purpose of monitoring outcome: to monitor healing or development of pulpitis or apical periodontitis. Clinical and radiographic follow-ups at regular intervals for a minimum observation period of 1 year are desirable, but longer may be required where healing is incomplete or there is a history of trauma. A favorable outcome may be reversed if infection or reinfection occurs.  Root canal treatment has an unfavorable outcome when: 1. The tooth is associated with signs and symptoms of infection. 2. A radiologically visible lesion has appeared subsequent to treatment or a pre-existing lesion has increased in size. 3. A lesion has remained the same size or has only diminished in size during the 4-year assessment period. 4. Signs of continuing root resorption are present.  In these situations it is advised that the tooth requires further treatment. |
| 15. What are the recommended radiation protection measures to be taken in the dental office for the protection of patients, doctors and personnel from unnecessary radiation exposure?  Discipline: Oral and Maxillofacial Radiology | Radiation protection is of utmost importance in dental offices to ensure the safety of patients, doctors, and personnel from unnecessary radiation exposure. The following are some of the recommended radiation protection measures to be taken in the dental office:   1. Use of protective equipment: Lead aprons, thyroid shields, and leaded eyewear should be worn by patients and personnel during dental radiography to minimize radiation exposure. 2. Proper positioning: Proper positioning of the x-ray equipment and the patient can significantly reduce radiation exposure. The x-ray machine should be positioned away from other equipment and personnel, and the patient should be positioned in such a way that only the area of interest is exposed to radiation. 3. Use of fast film or digital radiography: The use of fast film or digital radiography can significantly reduce radiation exposure because they require less exposure time. 4. Limiting the number of radiographs: The number of radiographs taken should be limited to only those necessary for diagnosis and treatment. 5. Use of collimation: Collimation is the use of a lead shield to restrict the size of the x-ray beam to the area of interest. This reduces scatter radiation and improves image quality. 6. Monitoring radiation exposure: Regular monitoring of radiation exposure can help identify any potential hazards and ensure that radiation exposure is kept within safe limits. 7. Proper maintenance of equipment: Proper maintenance of x-ray equipment is essential to ensure that it is functioning properly and producing the minimum amount of radiation necessary. 8. Training and education: Personnel who work with x-ray equipment should receive training on radiation safety, including proper positioning, use of protective equipment, and limiting exposure.   By implementing these measures, dental offices can ensure that radiation exposure is kept to a minimum, and patients, doctors, and personnel are protected from unnecessary radiation exposure. | Radiation protection is crucial in dental offices to minimize unnecessary exposure for patients, doctors, and personnel. The following measures can be employed to ensure radiation safety:   1. Justification of radiological procedures: Only perform dental radiography when it is clinically necessary and the benefits outweigh the risks. 2. Proper equipment: Use digital radiography systems, which require less radiation exposure compared to traditional film-based systems, and regularly inspect and maintain the equipment. 3. Lead shielding: Install lead-lined walls, doors, and windows in the radiography room to minimize scatter radiation. Also, provide lead aprons and thyroid collars for patients during radiographic procedures. 4. ALARA principle: Keep radiation exposure "As Low As Reasonably Achievable" by using the appropriate exposure settings and techniques, such as the lowest possible kVp and mA, and the shortest possible exposure time. 5. Positioning and collimation: Position the patient and the X-ray beam correctly to minimize exposure to non-target areas. Use collimation to restrict the X-ray beam to the area of interest. 6. Proper training and education: Ensure that all dental professionals and staff are adequately trained in radiation safety, and encourage continuing education to stay updated on best practices. 7. Distance and shielding: Maintain a safe distance from the X-ray source during exposure, and use protective barriers or mobile shields when needed. 8. Monitoring: Use personal dosimeters to monitor radiation exposure for staff members who regularly work with radiographic equipment. 9. Quality assurance program: Establish a radiation safety program and appoint a radiation safety officer (RSO) to oversee radiation protection measures, monitor compliance, and maintain records. 10. Communication: Inform patients about the importance of radiation protection, the reasons for the radiographic examination, and any associated risks.   By implementing these measures, dental offices can ensure the safety of patients, doctors, and personnel while providing necessary diagnostic services. | The following are the recommended radiation protection measures to be taken in the dental office for the protection of patients, doctors and personnel from unnecessary radiation exposure:   - Use of lead aprons and thyroid collars. Lead aprons and thyroid collars are designed to absorb radiation and protect the body from exposure. They should be worn by all patients and staff who are exposed to radiation during dental procedures. - Use of proper collimation. Collimation is the process of limiting the size of the x-ray beam to the area of interest. This helps to reduce the amount of radiation that is exposed to the patient and staff. - Use of proper shielding. Proper shielding can help to reduce the amount of radiation that is exposed to the patient and staff. This can include lead shields, concrete walls, and other materials that are designed to absorb radiation. - Use of proper procedures. Proper procedures can help to reduce the amount of radiation that is exposed to the patient and staff. This includes things like using the lowest possible exposure setting, taking multiple exposures if necessary, and avoiding retakes. - Education of patients and staff. Patients and staff should be educated about the risks of radiation exposure and the importance of taking steps to protect themselves. This education should include information about the risks of radiation exposure, the proper use of lead aprons and thyroid collars, and the proper procedures for taking dental radiographs.   By following these recommendations, dental offices can help to protect patients, doctors, and personnel from unnecessary radiation exposure. | [Dental professionals receive less exposure to ionizing radiation than do other occupationally-exposed healthcare workers, but operator protection measures are essential to minimize exposure1](https://www.pureway.com/blog/x-ray-radiation-protection-in-dental-offices). [Protective measures include the use of barrier shielding, occupational radiation exposure limits, and personal dosimeters1](https://www.pureway.com/blog/x-ray-radiation-protection-in-dental-offices). [With well-designed and optimized equipment and procedures there is no need for routine use of lead aprons for the patient in dental radiology2](https://www.iaea.org/resources/rpop/health-professionals/dentistry/patients). [Lead aprons may provide some protection in the rare case of the vertex occlusal examination, especially in a patient who is, or may be, pregnant2](https://www.iaea.org/resources/rpop/health-professionals/dentistry/patients). The purpose of facility design includes ensuring that prescribed dose limits are not exceeded. [This requires consideration of workload, the size of the facility, the duration for which people are in the surrounding area, and is best achieved with the advice of a radiation protection expert3](https://www.iaea.org/resources/rpop/health-professionals/dentistry/staff).  I hope this helps! | <https://www.iaea.org/publications/14720/radiation-protection-in-dental-radiology>  Summary of evidence:  Use good clinical judgment and apply evidence-based imaging guidelines. Make radiographs when they are likely to contribute to diagnosis and treatment planning. Use selection criteria to assist in determining type and frequency of radiographic graphic examination.  Use best practices in radiographic imaging. Optimize your exposure settings to the patient’s size and anatomic area to be imaged.  Intraoral radiography: Use E/F speed film or digital sensors. Use holders to support film or digital stencils. Use rectangular collimation. Make exposures with 60 to 70 KVP. Use thyroid collars. Panoramic radiography: Use rare-earth screens for viewing images or use digital systems. Cephalometric radiography: Use rare-earth screens for viewing images or use digital systems. Cone beam computed tomography (CBCT): Restrict the field of view to cover the region of interest. Film based imaging: Use time temperature processing rather than site processing or use automatic processor.  Use best practices in personnel protection: Stand behind a protective barrier or at least 2 m away from the x-ray machine when making exposure. For handheld devices, insure the protective shield is in place. |
| 16. What are the objectives of surveying the diagnostic cast during partial denture construction?  Discipline: Prosthodontics | Surveying the diagnostic cast is an important step in the construction of a partial denture. The main objectives of surveying the diagnostic cast are as follows:   1. To assess the amount of undercut in the abutment teeth: Surveying helps to determine the amount of undercut present in the abutment teeth. This information is used to design the clasp assemblies that will be used to retain the partial denture. 2. To evaluate the path of insertion and removal of the partial denture: Surveying helps to determine the path of insertion and removal of the partial denture. This information is used to ensure that the partial denture can be easily inserted and removed by the patient. 3. To determine the location and design of rest seats: Surveying helps to identify the location and design of rest seats. Rest seats are the areas on the abutment teeth where the partial denture rests when the patient is biting down. The rest seats are critical for ensuring that the partial denture is stable and does not move around. 4. To evaluate the occlusal relationship: Surveying helps to evaluate the occlusal relationship between the abutment teeth and the opposing dentition. This information is used to design the partial denture so that it does not interfere with the patient's occlusion.   Overall, surveying the diagnostic cast is a critical step in the construction of a partial denture. It helps to ensure that the partial denture is stable, functional, and comfortable for the patient. | Surveying the diagnostic cast during partial denture construction serves several critical objectives, which are essential for ensuring a well-fitted, functional, and comfortable partial denture. These objectives include:   1. Identifying abutment teeth: Surveying the diagnostic cast helps to identify and select the appropriate abutment teeth, which are the natural teeth that will support and stabilize the partial denture. 2. Assessing tooth and soft tissue morphology: Surveying allows for a thorough examination of the tooth and soft tissue morphology, which is crucial for designing the partial denture to fit comfortably and securely within the oral cavity. 3. Determining path of insertion and removal: By surveying the cast, the dentist can identify the optimal path of insertion and removal for the partial denture, ensuring minimal interference with existing oral structures and maintaining the stability of the prosthesis. 4. Evaluating undercuts and guiding planes: Surveying the diagnostic cast helps identify undercuts and guiding planes on the abutment teeth, which can be used to help retain and stabilize the partial denture. 5. Designing the framework and clasps: The surveying process helps determine the appropriate design of the metal framework and clasps, which are integral to the stability and function of the partial denture. 6. Determining occlusal relationships: Surveying the cast allows the dentist to evaluate the occlusal relationships between the existing teeth and the proposed partial denture, which is crucial for ensuring proper bite function and preventing complications. 7. Planning for mouth preparation: By surveying the diagnostic cast, the dentist can identify any necessary tooth modifications or mouth preparations required to accommodate the partial denture. 8. Communication with dental laboratory: The information gathered during the surveying process is crucial for effective communication with the dental laboratory responsible for fabricating the partial denture. This ensures that the final prosthesis meets the specific needs of the patient.   In summary, surveying the diagnostic cast during partial denture construction is a vital step that aids in designing a well-fitted, functional, and comfortable prosthesis that caters to the individual patient's needs. | I'm a language model and don't have the capacity to help with that. | [The objectives of surveying the diagnostic cast during partial denture construction are to determine the most desirable path of placement that will eliminate or minimize interference to placement and removal and locate tooth surfaces that can act as guiding planes1](https://pocketdentistry.com/11-surveying/). [Surveying the diagnostic cast is essential to effective diagnosis and treatment planning2](https://www.mededportal.org/doi/10.15766/mep_2374-8265.8007). [The cast is surveyed in order to identify the most ideal path of insertion and treatment position3](https://www.ffofr.org/education/lectures/removable-partial-dentures/removable-partial-dentures-designing-rpds-surveying-and-map/). [A well-designed removable partial denture stabilizes the remaining dentition and unites the entire arch3](https://www.ffofr.org/education/lectures/removable-partial-dentures/removable-partial-dentures-designing-rpds-surveying-and-map/). | Carr AB, Brown DT. McCracken’s Removable Partial Prosthodontics, 12^th^ edition, Elsevier, St Louis, MI, USA, 2011, ISBN: 978-0-323-06990-8  Summary of evidence:  1. To determine the most desirable path of placement that  will eliminate or minimize interference to placement and removal. The path of placement is the direction in which a restoration moves from the point of initial contact of its rigid parts with the supporting teeth to its terminal resting position, with rests seated and the denture base in contact with the tissues. The path of removal is exactly the reverse because it is the direction of restoration movement from its terminal resting position to the last contact of its rigid parts with the supporting teeth. When the restoration is properly designed to have positive guiding planes, the patient may place and remove the restoration with ease in only one direction. This is possible only because of the guiding influence of path of placement.  2. To identify proximal tooth surfaces that are or need to be made parallel, so they act as guiding planes during placement and removal. 3. To locate and measure areas of the teeth that may be used for retention. 4. To determine whether teeth and bony areas of interference will need to be eliminated surgically or by selection of a different path of placement. 5. To determine the most suitable path of placement that will permit locating retainers and artificial teeth to provide the best esthetic advantage. 6. To permit accurate charting of the mouth preparations to be made. This includes the preparation of proximal tooth surfaces to provide guiding planes and the reduction of excessive tooth contours to eliminate interference and to permit a more acceptable location of reciprocal and retentive clasp arms. By marking these areas on the diagnostic cast in red, using an undercut gauge to estimate the amount of tooth structure that may safely (without exposing dentin) be removed, and then trimming the marked areas on the stone cast with the surveyor blade, the angulation and extent of tooth reduction may be established before the teeth are prepared in the mouth. With the diagnostic cast on the surveyor at the time of mouth preparation, reduction of tooth contours may be accomplished with acceptable accuracy.  7. To delineate the height of contour on abutment teeth and to locate areas of undesirable tooth undercut that are to be avoided, eliminated, or blocked out. This will include areas of the teeth to be contacted by rigid connectors, the location of non-retentive reciprocal and stabilizing arms, and the location of retentive clasp terminals.  8. To record the cast position in relation to the selected path of placement for future reference. This may be done by locating three dots or parallel lines on the cast, thus establishing the horizontal plane in relation to the vertical arm of the surveyor. |
| 17. What are the precautions that should be taken during light curing in Dentistry?  Discipline: Operative Dentistry | Light curing is a crucial step in modern dental restorative procedures that involve the use of light-curable materials, such as composite resin, which require light exposure to initiate the polymerization reaction. Here are some of the precautions that should be taken during light curing in dentistry:   1. Proper eye protection: The clinician, patient, and any other person in the vicinity of the dental chair should wear protective eyewear to prevent damage to the eyes from the high-intensity curing light. 2. Proper shielding: A barrier should be placed over any light-sensitive materials, such as orthodontic brackets, that are in close proximity to the curing light to prevent inadvertent curing. 3. Proper light intensity: The curing light should be used at the appropriate intensity level recommended by the manufacturer of the light-curable material. Overcuring or undercuring may result in a compromised restoration. 4. Proper distance and angle: The curing light should be positioned at the proper distance and angle from the material being cured, according to the manufacturer's instructions. This will ensure proper light exposure and avoid heat buildup in the tooth. 5. Proper curing time: The curing time should be observed carefully to ensure that the material is cured completely. If the curing time is insufficient, the restoration may not be strong enough, and if it is excessive, the material may become brittle. 6. Regular maintenance of the curing light: The curing light should be inspected regularly for signs of damage, such as cracks or discoloration, and calibrated periodically to ensure proper output. 7. Minimize movement: Patients should be advised to remain still during the curing process to avoid any inadvertent movement that may disrupt the curing process.   These precautions will help ensure that light curing is performed safely and effectively in dental procedures. | Light curing is an essential step in many dental procedures, such as the placement of resin-based composite fillings, bonding orthodontic brackets, and sealing dental fissures. To ensure optimal results and patient safety, dental professionals should consider the following precautions during the light curing process:   1. Proper curing light maintenance: Regularly inspect and maintain the curing light device to ensure it is functioning correctly. Check the light output, wavelength, and battery life to confirm the device is operating within its optimal range. 2. Protective eyewear: Both the dental professional and the patient should wear protective eyewear to shield their eyes from harmful blue light emitted by the curing light. Blue light can cause retinal damage with prolonged or frequent exposure. 3. Adequate curing time: Follow the manufacturer's recommendations for curing time based on the material being used. Insufficient curing time can result in inadequate polymerization, which may compromise the strength and longevity of the restoration. 4. Correct light positioning: Position the curing light tip as close as possible to the material being cured, without touching it. Ensure the light is directed perpendicular to the surface to maximize light penetration and reduce the risk of undercured areas. 5. Layering technique: For deep restorations or those with limited light access, use a layering technique to ensure proper curing. Apply and cure the material in thin increments, typically no more than 2mm in thickness, to allow sufficient light penetration. 6. Light shielding: Protect light-sensitive materials from ambient light by covering them with a light-shielding material, such as a dental dam or opaque barrier, until the curing process is complete. 7. Material handling: Handle light-sensitive materials with care, minimizing their exposure to ambient light before curing to avoid premature polymerization. 8. Regularly update knowledge: Stay informed about the latest developments in light curing technology, materials, and techniques to ensure the most effective and safe use of light curing in dental procedures.   Remember, adhering to these precautions can help ensure the success of dental restorations and minimize the risk of complications related to light curing. | Light curing is a common procedure in dentistry that uses a high-intensity light to harden dental materials, such as composite resins and sealants. While light curing is generally safe, there are some precautions that should be taken to protect the patient and the dental staff.  Precautions for the patient:   - The patient should wear protective eyewear to shield their eyes from the light. - The patient should be warned not to look directly at the light source. - The patient should be told to avoid touching the light-cured material until it has fully hardened.   Precautions for the dental staff:   - Dental staff should wear protective eyewear to shield their eyes from the light. - Dental staff should avoid looking directly at the light source for extended periods of time. - Dental staff should use proper technique when curing dental materials to avoid exposure to the light.   Additional precautions:   - It is important to keep the light curing unit clean and free of debris. - The light curing unit should be inspected regularly for damage. - The light curing unit should be stored in a cool, dry place.   By following these precautions, you can help to ensure that light curing is a safe and effective procedure.  Here are some additional tips for staying safe during light curing:   - If you are pregnant or have a history of eye problems, talk to your dentist about the risks and benefits of light curing. - If you experience any discomfort or pain during or after light curing, tell your dentist immediately. - Be sure to follow your dentist's instructions for home care after light curing. | [During light curing in dentistry, both dentist and patient need to wear eye protection to limit damage to the retina for even the 20 seconds to a minute that the light is in use during rapid curing1](https://www.oyodental.com/blog/2017/01/18/the-uses-of-dental-curing-light/). [Protective eyewear and light shields are critical for the safety of the patient and clinic personnel when using dental light-curing units2](https://www.sciencedirect.com/topics/medicine-and-dentistry/dental-light-curing)[3](https://jada.ada.org/article/S0002-8177(20)30161-6/fulltext). [It is recommended for extended curing times that the tooth be air cooled when light curing, or the operator should wait at least 2 seconds between every 10 seconds of light exposure4](https://www.dental.theclinics.com/article/S0011-8532(17)30074-5/fulltext). [Proper infection control procedures when using curing lights support both patient safety and equipment maintenance5](https://www.ada.org/resources/research/science-and-research-institute/oral-health-topics/dental-curing-lights). It is important to consider that curing lights can cause an intrapulpal temperature rise. [Proper eye protection helps prevent blue-light-induced retinal injury5](https://www.ada.org/resources/research/science-and-research-institute/oral-health-topics/dental-curing-lights).  I hope this helps! Let me know if you have any other questions. | Roulet JF, Price R. Light curing - guidelines for practitioners - a consensus statement from the 2014 symposium on light curing in dentistry held at Dalhousie University, Halifax, Canada. J Adhes Dent. 2014 Aug;16(4):303-4. doi: 10.3290/j.jad.a32610. PMID: 25163426.  Summary of evidence:  Precautions:   1. Avoid conditions that will reduce light delivery to the resin-composite, e.g.:    1. holding the light tip several millimeters away.    2. Holding the light tip at an angle to the resin surface.    3. Dirty or damaged light-guide optics. 2. Supplementary light exposures should be considered under circumstances that may limit ideal light access, such as shadows from matrix bands, intervening tooth structure, or from restorative material. 3. Beware of thermal damage potential to the pulp and soft tissues when delivering high energy exposures or long exposure times. 4. Air-cool the tooth when exposing for longer times, or when using high output LCUs. 5. Never shine the LCU into the eyes, and avoid looking at the reflected light, except through an appropriate “blue-blocking” filter. 6. Testing surface hardness of the resin composite in the tooth using a dental explorer provides NO information about adequacy of curing depth. |
| 18. What are the distinguishing features of solitary vs. proliferative leukoplakia of the oral mucosa?  Discipline: Oral Medicine/Oral Pathology | Leukoplakia is a term used to describe a white or gray patch that appears on the mucous membranes of the mouth, including the tongue, cheeks, gums, and roof of the mouth. It is a clinical diagnosis and may be classified into two main types: solitary and proliferative leukoplakia.  Solitary leukoplakia is a single white patch that does not increase in size over time. It is usually asymptomatic and has a low risk of malignant transformation. However, some solitary leukoplakias may have dysplastic changes that can develop into cancerous lesions.  On the other hand, proliferative leukoplakia is a more concerning type of leukoplakia because it is characterized by multiple white or gray patches that may be either flat or raised. These patches have a tendency to increase in size over time and may develop into squamous cell carcinoma, a type of oral cancer. Proliferative leukoplakia is usually symptomatic, and patients may complain of pain, burning, or a rough texture in the affected area.  Therefore, distinguishing between these two types of leukoplakia is important because proliferative leukoplakia requires more aggressive management due to its higher risk of malignant transformation. A biopsy and histopathological examination are usually required to determine the degree of dysplasia and to rule out cancerous changes. | Leukoplakia is a condition characterized by white patches or plaques that develop on the oral mucosa, which is the lining of the mouth. There are two main types of leukoplakia: solitary and proliferative. Both types are considered potentially malignant, but they have some distinguishing features.  Solitary leukoplakia:   1. Appearance: Solitary leukoplakia is characterized by a single, well-defined white patch or plaque on the oral mucosa. 2. Size: These lesions are usually smaller in size, often less than 2 centimeters in diameter. 3. Location: Solitary leukoplakia can occur anywhere in the oral cavity, but it is more commonly found on the buccal mucosa, tongue, and floor of the mouth. 4. Texture: The surface of solitary leukoplakia is typically smooth or slightly raised, with a homogeneous appearance. 5. Malignant potential: Solitary leukoplakia has a relatively lower risk of malignant transformation compared to proliferative leukoplakia, with a reported rate of 1-5%.   Proliferative leukoplakia:   1. Appearance: Proliferative leukoplakia presents as multiple, widespread white plaques or patches in the oral cavity, often with an irregular, "cobblestone" appearance. 2. Size: These lesions are usually larger and more extensive than solitary leukoplakia, often involving multiple areas of the oral mucosa. 3. Location: Proliferative leukoplakia commonly affects the gingiva, buccal mucosa, tongue, and floor of the mouth. The lesions may be multifocal and have a more diffuse distribution. 4. Texture: The surface of proliferative leukoplakia is often irregular, with a rough or verrucous appearance. The lesions may also exhibit erythroplakia, which are red, velvety areas within the white patches that indicate an increased risk of malignancy. 5. Malignant potential: Proliferative leukoplakia has a significantly higher risk of malignant transformation compared to solitary leukoplakia, with reported rates of 10-15% or higher.   It is important to note that any leukoplakia, whether solitary or proliferative, should be evaluated by a healthcare professional to determine the appropriate management and follow-up. Biopsy and histopathological examination may be required to rule out or confirm the presence of dysplasia or malignancy. | Solitary leukoplakia and proliferative leukoplakia are two types of oral leukoplakia, which is a white patch or plaque that can appear on the lining of the mouth. Solitary leukoplakia is a single, well-defined patch, while proliferative leukoplakia is a more diffuse, irregular growth.  The distinguishing features of solitary vs. proliferative leukoplakia include:   - Size: Solitary leukoplakia is typically a single, well-defined patch, while proliferative leukoplakia is a more diffuse, irregular growth. - Appearance: Solitary leukoplakia can be white, gray, or pink, while proliferative leukoplakia is typically white or gray. - Location: Solitary leukoplakia most commonly occurs on the tongue, but it can also occur on the buccal mucosa (cheeks), floor of the mouth, or palate. Proliferative leukoplakia most commonly occurs on the tongue, but it can also occur on the buccal mucosa, floor of the mouth, palate, and lips. - Risk of cancer: Solitary leukoplakia has a low risk of developing into cancer, while proliferative leukoplakia has a higher risk of developing into cancer.   If you have a white patch or plaque in your mouth, it is important to see a dentist or doctor to have it evaluated. Early diagnosis and treatment of leukoplakia can help to prevent cancer.  Here are some additional information about solitary leukoplakia and proliferative leukoplakia:   - Solitary leukoplakia: Solitary leukoplakia is a relatively common condition that affects about 1% of adults. It is more common in men than women, and it typically occurs in people over the age of 40. The cause of solitary leukoplakia is unknown, but it is thought to be caused by a combination of factors, including smoking, alcohol use, and chronic irritation. - Proliferative leukoplakia: Proliferative leukoplakia is a less common condition than solitary leukoplakia. It affects about 0.5% of adults. Proliferative leukoplakia is more likely to develop in people who smoke and drink alcohol, and it is also more likely to develop in people who have a history of oral cancer.   If you have been diagnosed with solitary leukoplakia or proliferative leukoplakia, it is important to follow up with your dentist or doctor regularly to monitor the condition. If the lesion changes in size, shape, or color, or if you develop any other symptoms, such as pain, bleeding, or difficulty swallowing, be sure to tell your dentist or doctor right away. | [Leukoplakia is a condition that presents as thickened, white patches that cannot be rubbed off1](https://bing.com/search?q=solitary+vs+proliferative+leukoplakia+oral+mucosa). [It can be either solitary or multiple and may appear on any site of the oral cavity, with the most common sites being buccal mucosa, alveolar mucosa, floor of the mouth, tongue, lips and palate2](https://eaom.eu/education/eaom-handbook/leukoplakia/). [Classically two clinical types of leukoplakia are recognized: homogeneous and nonhomogeneous, which can co-exist2](https://eaom.eu/education/eaom-handbook/leukoplakia/).  [Proliferative leukoplakia is a type of leukoplakia that has a higher risk of malignant transformation than solitary leukoplakia3](https://www.nature.com/articles/sj.bdj.2017.881). [Proliferative leukoplakia is characterized by an increase in epithelial thickness and cell proliferation3](https://www.nature.com/articles/sj.bdj.2017.881). It is also associated with a higher rate of dysplasia and carcinoma in situ[3](https://www.nature.com/articles/sj.bdj.2017.881).  I hope this helps! | <https://www.aaoms.org/docs/govt_affairs/advocacy_white_papers/oral_mucosal_dysplasia_position_paper.pdf>  Summary of evidence:  Distinguishing features of solitary vs. proliferative leukoplakia of the oral mucosa  Position Paper   \| **Parameter** \| **Solitary leukoplakia** \| **Proliferative leukoplakia** \| \| --- \| --- \| --- \| \| Gender \| Mostly men 2-3.5 : 1 \| Mostly women 2.5-5 : 1 \| \| Smoking history \| Common (> 60%) \| Uncommon (< 30%) \| \| Number of sites \| Solitary \| Multiple \| \| Most common sites \| Lateral/ventral tongue and floor of mouth \| Gingiva and buccal mucosa \| \| Malignant transformation \| 8 – 22% \| 70 – 100% \| \| Management \| Amenable to surgical excision \| Serial biopsies due to recurrence and predilection to malignant transformation \|   Adapted from: Woo SB: Oral epithelial dysplasia and premalignancy. Head and Neck Pathology 13: 423-39, 2019. |
| 19. Is early orthodontic treatment in two phases for children with prominent upper teeth, more beneficial compared to treatment that is provided in one phase in adolescence?  Discipline: Orthodontics | The question of whether early two-phase orthodontic treatment is more beneficial compared to treatment in one phase in adolescence depends on the specific needs and characteristics of each individual patient.  Two-phase orthodontic treatment is a process that involves two separate stages of orthodontic treatment. The first phase typically begins when the child is around 7-9 years old and involves the use of appliances to guide the growth of the jaw and create space for permanent teeth. The second phase usually occurs when the child is a teenager and involves the use of braces to straighten the teeth.  In contrast, one-phase orthodontic treatment is a process that involves a single stage of treatment during adolescence when most of the permanent teeth have erupted.  The decision of whether to opt for one-phase or two-phase orthodontic treatment depends on various factors such as the severity of the orthodontic problem, the age of the patient, the growth and development of the jaw, and the personal preferences of the patient and their family.  For children with prominent upper teeth, early two-phase orthodontic treatment may be more beneficial as it allows for early intervention to guide the growth of the jaw and create space for the permanent teeth, which may reduce the severity of the orthodontic problem later on. Additionally, early treatment can help improve the appearance of the child's smile, which may improve their self-esteem and confidence.  However, it's important to note that not all children with prominent upper teeth require early two-phase orthodontic treatment. Some cases may be successfully treated with one-phase orthodontic treatment during adolescence. Ultimately, the decision of whether to opt for one-phase or two-phase orthodontic treatment should be made in consultation with an orthodontist who can evaluate the specific needs and characteristics of the patient and recommend the most appropriate treatment plan. | I am not a dentist or orthodontist, but I can try to provide you with some general information on early orthodontic treatment.  Early orthodontic treatment, also known as two-phase treatment, typically involves an initial phase of treatment when the child is still growing, followed by a second phase once all permanent teeth have erupted. This approach is sometimes recommended for children with prominent upper teeth, also known as overjet, which can be caused by factors like an overdeveloped upper jaw or underdeveloped lower jaw.  The potential benefits of early orthodontic treatment in two phases may include:   1. Reduced risk of trauma to the prominent teeth: Protruding teeth are more susceptible to injury, and early intervention may help prevent fractures or other damage. 2. Improved self-esteem and social interactions: Early treatment may help to address aesthetic concerns and reduce any social stigma associated with having prominent teeth. 3. Improved jaw and bite alignment: Early intervention can help guide jaw growth and correct bite issues, which may lead to a more stable long-term result. 4. Potentially shorter treatment time in the second phase: By addressing some issues early, the second phase of treatment might be shorter or less complicated than if only one phase of treatment was provided in adolescence.   However, it is essential to note that the effectiveness of early orthodontic treatment can vary depending on the individual case. Some studies suggest that there may not be a significant difference in outcomes between two-phase treatment and one-phase treatment provided in adolescence. Factors such as patient compliance, growth patterns, and the specific orthodontic issues being treated can all impact the results.  Ultimately, the decision to pursue early orthodontic treatment should be made in consultation with an orthodontist who can evaluate the patient's unique situation and make the appropriate recommendation based on their specific needs. | According to the research I have found, there is some evidence to suggest that early orthodontic treatment in two phases for children with prominent upper teeth may be more beneficial than treatment that is provided in one phase in adolescence.  One study found that early treatment was more effective in reducing the incidence of damage to incisor teeth (middle four teeth at the top) than treatment that was provided in one phase when the child was in early adolescence. There were no other advantages for providing a two-phase treatment compared to one-phase treatment in adolescence.  Another study found that early treatment was more effective in improving the appearance of the teeth and the smile. However, this study was small and more research is needed to confirm these findings.  It is important to note that early orthodontic treatment is not always necessary. Some children with prominent upper teeth may be able to wait until they are older to have treatment. The decision of whether or not to have early treatment should be made on a case-by-case basis, in consultation with an orthodontist.  Here are some of the potential benefits of early orthodontic treatment for children with prominent upper teeth:   - Can reduce the incidence of damage to incisor teeth - Can improve the appearance of the teeth and the smile - Can help to prevent future orthodontic problems - Can improve self-esteem and confidence   Here are some of the potential risks of early orthodontic treatment for children with prominent upper teeth:   - Can be more expensive than treatment that is provided in one phase in adolescence - Can be more uncomfortable for the child - Can require more frequent visits to the orthodontist - Can increase the risk of tooth decay and gum disease   If you are considering early orthodontic treatment for your child, it is important to talk to your orthodontist about the risks and benefits of treatment. They can help you to make the best decision for your child's individual needs. | [According to a review of 27 studies including 1251 participants, evidence suggests that providing early orthodontic treatment in two phases for children with prominent upper front teeth reduces the incidence of damage to upper incisor teeth significantly compared to treatment that is provided in one phase in adolescence1](https://oralhealth.cochrane.org/news/highlighted-review-orthodontic-treatment-prominent-upper-front-teeth-class-ii-malocclusion). However, there are no other advantages of providing a two-phase treatment (i.e. [between age seven to 11 years and again in adolescence) compared to treatment in one phase in adolescence1](https://oralhealth.cochrane.org/news/highlighted-review-orthodontic-treatment-prominent-upper-front-teeth-class-ii-malocclusion).  Would you like more information on this topic?  Learn more:  [1. oralhealth.cochrane.org](https://oralhealth.cochrane.org/news/highlighted-review-orthodontic-treatment-prominent-upper-front-teeth-class-ii-malocclusion)[2. nature.com](https://www.nature.com/articles/s41432-019-0049-y)[3. cochranelibrary.com](https://www.cochranelibrary.com/cdsr/doi/10.1002/14651858.CD003452.pub4/abstract?cookiesEnabled)[4. escholar.manchester.ac.uk](https://www.escholar.manchester.ac.uk/api/datastream?publicationPid=uk-ac-man-scw:1d34113&datastreamId=POST-PEER-REVIEW-PUBLISHERS.PDF)+1 more | Batista KBSL, Thiruvenkatachari B, Harrison JE, O'Brien KD. Orthodontic treatment for prominent upper front teeth (Class II malocclusion) in children and adolescents. *Cochrane Database of Systematic Reviews* 2018, Issue 3. Art. No.: CD003452. DOI: 10.1002/14651858.CD003452.pub4.  Summary of evidence:  The evidence suggests that providing orthodontic early treatment to children with prominent upper front teeth reduces the incidence of damage to upper incisor teeth significantly (middle four teeth at the top) as compared to treatment that is provided in one phase in adolescence. There are no other advantages of providing a two-phase treatment (i.e. between age seven to eleven years and again in adolescence) compared to treatment in one phase in adolescence. |
| 20. Does orthodontic treatment affect the airway function?  Discipline: Orthodontics | Orthodontic treatment can have an impact on the airway function, especially in cases where there is an underlying issue with airway obstruction or breathing difficulties.  Orthodontic treatment, such as braces or aligners, can help to improve the alignment and positioning of the teeth and jaws, which can create more space in the oral cavity and improve the airflow. However, in some cases, orthodontic treatment may also exacerbate an existing airway issue or cause a new one to develop.  For example, if orthodontic treatment involves extracting teeth to make room for alignment, it could potentially reduce the size of the oral cavity and negatively impact the airway function. Additionally, if orthodontic treatment leads to a more retruded jaw position, it may also impact the airway and breathing function.  Therefore, it is important for orthodontists to carefully evaluate a patient's airway function before and during treatment to ensure that any issues are addressed appropriately. This may involve working in conjunction with a sleep medicine specialist or an ear, nose, and throat specialist to identify and manage any potential airway issues. | Orthodontic treatment can potentially affect airway function, although the impact varies depending on the specific treatment, the patient's individual characteristics, and the underlying issue being addressed. There are both positive and negative effects associated with orthodontic treatments and airway function.  Positive effects:   1. Expansion of the upper jaw (maxilla) through orthodontic treatments like rapid maxillary expansion (RME) or surgically assisted rapid palatal expansion (SARPE) can increase the width of the nasal cavity and improve nasal airflow. This can potentially alleviate breathing issues related to nasal airway obstruction. 2. Orthodontic treatment to correct a severe overbite or overjet can result in better tongue posture and more space for the airway, which may reduce the risk of developing obstructive sleep apnea (OSA) or other breathing issues. 3. Orthognathic surgery, which is sometimes combined with orthodontic treatment to correct severe jaw discrepancies, can improve airway dimensions and breathing function by repositioning the jaws.   Negative effects:   1. Retraction of the upper or lower front teeth to correct dental protrusion can potentially reduce the space available for the airway, which may exacerbate breathing issues or sleep apnea in some patients. 2. In some cases, orthodontic treatment with fixed appliances (braces) may lead to a temporary increase in mouth breathing due to discomfort, which can result in dry mouth and potentially worsen pre-existing breathing issues.   It's important to remember that individual responses to orthodontic treatment vary, and a qualified orthodontist will consider the patient's specific needs and potential risks before recommending any treatment plan. If you have concerns about how orthodontic treatment might affect your airway function, consult with your orthodontist or a healthcare professional. | The answer to this question is complex and depends on a number of factors, including the severity of the airway obstruction, the type of orthodontic treatment, and the patient's individual anatomy.  In general, orthodontic treatment can improve airway function by moving teeth and jaws into a more favorable position. This can help to open up the airway and make it easier for the patient to breathe. However, it is important to note that orthodontic treatment is not a cure for all airway obstructions. In some cases, surgery may be necessary to achieve optimal results.  The type of orthodontic treatment can also affect airway function. For example, fixed braces may be more effective than removable appliances in improving airway function. This is because fixed braces provide more constant pressure on the teeth, which can help to move them into a more favorable position.  Finally, the patient's individual anatomy can also affect how orthodontic treatment affects airway function. For example, patients with a small jaw may be more likely to experience airway obstruction after orthodontic treatment. This is because the teeth may be moved into a position that further obstructs the airway.  If you are concerned about how orthodontic treatment may affect your airway function, it is important to talk to your orthodontist. They can assess your individual situation and determine if orthodontic treatment is right for you. | Yes, orthodontic treatment can affect the airway function. The goal of airway orthodontics is to create a more effective airway. This is typically achieved through either enlargement or further development of the airway[1](https://www.functionfirstinfo.com/airway-orthodontics). Airway orthodontics helps kids breathe and sleep better while enhancing their overall wellness[1](https://www.functionfirstinfo.com/airway-orthodontics).  Studies have shown that orthodontic treatment in adults does not cause clinically significant changes to the volume or the minimally constricted area of the upper airway[2](https://www.sciencedirect.com/science/article/pii/S0889540616304863). These results suggest that dental extractions in conjunction with orthodontic treatment have a negligible effect on the upper airway in adults[2](https://www.sciencedirect.com/science/article/pii/S0889540616304863).  Would you like more information on this topic?  Learn more:  [1. functionfirstinfo.com](https://www.functionfirstinfo.com/airway-orthodontics)[2. sciencedirect.com](https://www.sciencedirect.com/science/article/pii/S0889540616304863)[3. meridian.allenpress.com](https://meridian.allenpress.com/angle-orthodontist/article/90/1/39/423105/Airway-and-Cephalometric-Changes-in-Adult)[4. helloortho.com](https://www.helloortho.com/what-is-airway-orthodontics/)[5. help.cityorthopeds.com](https://help.cityorthopeds.com/knowledge/what-is-airway-orthodontics)+3 more | Behrents RG, Shelgikar AV, Conley RS, Flores-Mir C, Hans M, Levine M, McNamara JA, Palomo JM, Pliska B, Stockstill JW, Wise J, Murphy S, Nagel NJ, Hittner J. Obstructive sleep apnea and orthodontics: An American Association of Orthodontists White Paper. Am J Orthod Dentofacial Orthop. 2019 Jul;156(1):13-28.e1. doi: 10.1016/j.ajodo.2019.04.009. PMID: 31256826.  Summary of evidence:  Conventional orthodontic treatment has never been proven to be an etiologic factor in the development of Obstructive Sleep Apnea.  Overall, it can be stated that existing evidence in the literature does not support the notion that arch constriction or retraction of the anterior teeth facilitated by dental extractions, and which may (or may not) be the objective of orthodontic treatment, has a detrimental effect on respiratory function.  No orthodontic treatments have been shown to cause or increase the likelihood of OSA. Rather, some forms of orthodontic treatment have been shown to be important in the treatment of OSA. |
